# Taxonomic revision of *Martinella* Baill. (Bignonieae, Bignoniaceae)

**DOI:** 10.3897/phytokeys.177.64465

**Published:** 2021-05-13

**Authors:** Eric Y. Kataoka, Lúcia G. Lohmann

**Affiliations:** 1 Laboratório de Sistemática Vegetal, Departamento de Botânica, Instituto de Biociências, Universidade de São Paulo, Rua do Matão, 277, 05508-090, São Paulo, SP, Brazil Universidade de S&atilde;o Paulo S&atilde;o Paulo Brazil

**Keywords:** Amazon, lianas, Neotropical flora, taxonomy

## Abstract

*Martinella* Baill. is a genus of Neotropical lianas in tribe Bignonieae (Bignoniaceae). The genus is monophyletic and well supported by morphological and molecular characters. Members of *Martinella* are characterized by a continuous interpetiolar ridge surrounding the stem, bilobed or 4–5-parted calyces, and minute triangular prophylls of the axillary buds. Generic circumscription remained unchanged since the description of the genus, although unclear species limits remained. Based on extensive fieldwork, herbarium work, and a molecular phylogenetic hypothesis for the genus, we here recognize five species of *Martinella*. Of these, three were recognized in earlier treatments for the genus, while two represent new species described here, *Martinella lanuginosa* Kataoka & L.G.Lohmann, **sp. nov.** and *Martinella tomentosa* Kataoka & L.G.Lohmann, **sp. nov.***Martinella iquitoensis* A.Samp. is treated as a synonym of *M. insculpta* Sprague & Sandwith. In addition, one second-step lectotype is designated for *Bignonia martini* DC., and neotypes are designated for *Doxantha longisiliqua* Miers and *Martinella gollmeri* K.Schum. This work provides a full taxonomic treatment for *Martinella*, including a complete list of synonyms, morphological descriptions, illustrations, photographs, distribution maps, conservation status, and comments for all five species recognized.

## Introduction

*Martinella* Baill. (Bignonieae, Bignoniaceae) is a genus of Neotropical lianas, whose monophyly is supported by molecular phylogenetic studies (Lohmann 2006) and by morphological features. Namely, minute triangular prophylls of the axillary buds and interpetiolar ridges surrounding the stems are thought to represent morphological synapomorphies of the genus (Lohmann and Taylor 2014). Additionally, silvery or whitish leaflets on the abaxial surface, bilobed or 4–5-parted calyces, and corollas with a constricted basal portion and an upper campanulate portion also characterize species of *Martinella*.

Baillon (1888) described *Martinella* based on reproductive characters such as the irregularly 2–4-lobed calyces, bilabiate corollas with a wide tube (presumably referring to what was named *Martinella*-type flower nearly a century later; see Gentry 1974), stipitate ovary that sits on a large nectariferous disk, and glabrous, flattened and narrow fruits. The genus was described based on *Bignonia martini* DC. [= *Martinella obovata* (Kunth) Bureau & K.Schum.], and the new combination *Martinella martini* (DC.) Baill. was proposed. Schumann (1894) described the new species, *Martinella gollmeri* K.Schum. [= *Martinella obovata*] based on the shallow and bowl-shaped nectariferous disk and frizzy calyces. Two years later, Bureau and Schumann (1896) transferred *Spathodea obovata* Kunth into *Martinella*, proposing the new combination *Martinella obovata* (Kunth) Bureau & K.Schum., which became the accepted name for *Martinella martini*.

Sprague and Sandwith (1934) described *Martinella insculpta* Sprague & Sandwith, while Sampaio (1935) described *M. iquitoensis* A.Samp. and *M. manaosiana* A.Samp. (Sampaio 1936); both of these names are synonyms of *M. insculpta*. More recently, Zuntini and Lohmann (2014) published a new species endemic to the Atlantic Forest, *M. insignis* A.H.Gentry ex Zuntini & L.G.Lohmann, expanding the distribution of this predominantly Amazonian genus.

The circumscription of *Martinella* remained very stable over the years (Lohmann and Taylor 2014). Despite the generic stability, delimitation of the Amazonian species remained confusing, which led some authors to consider the Amazonian *Martinella* as part of a species complex due to overlapping morphological characters (e.g., MacBride 1961, Zuntini and Lohmann 2014). Alwyn Gentry treated *M. insculpta* as a synonym of *M. obovata* in the floristic treatments of several Neotropical countries such as Panama (Gentry 1973), Ecuador (Gentry 1977), and Venezuela (Gentry 1982).

In the most recent synopsis of the genus (Zuntini and Lohmann 2014), three species were recognized: *M. insignis*, *M. iquitoensis* [= *M. insculpta*], and *M. obovata*. A recent molecular phylogeny of *Martinella* (Kataoka and Lohmann in prep.) recovered five main clades that are easily diagnosed by morphological features. Each of these clades is here recognized as a distinct species. Of these, three correspond to species treated in the recent synopsis of the genus (Zuntini and Lohmann 2014), i.e., *Martinella insculpta*, *Martinella insignis*, and *Martinella obovata*, while two correspond to taxa that are newly described here, i.e., *Martinella lanuginosa* Kataoka & L.G.Lohmann and *Martinella tomentosa* Kataoka & L.G.Lohmann.

## Methods

The taxonomic revision of *Martinella* was based on living specimens and observations made on fresh material during field expeditions and the analyses of herbarium specimens deposited in the following herbaria: IAN, INPA, MG, MO, NY, QCA, QCNE, R, RB, SPF, and UFACPZ (acronyms follow Thiers, continuously updated). All specimens were examined either physically or digitally through high quality photographs of herbarium specimens. In many cases we accessed specimens’ photographs that were available in virtual herbaria via the JSTOR Global Plants (https://plants.jstor.org/), the Reflora Virtual Herbarium (http://reflora.jbrj.gov.br/reflora/herbarioVirtual/), or websites of individual herbaria.

Field expeditions were conducted between June and December 2016 in the Brazilian states of Acre, Amazonas, Mato Grosso, Pará, and Roraima. All specimens were deposited at SPF. All accepted names are listed in alphabetical order. Nomenclatural discussions follow the International Code of Nomenclature for algae, fungi, and plants (Shenzhen Code) (Turland et al. 2018). Citations of type specimens are followed by the herbarium acronym and barcode, unless otherwise stated between brackets.

We used a molecular phylogeny (Kataoka and Lohmann in prep.) and morphological data to circumscribe species. We treated separately evolving lineages as species, following De Queiroz (2007). In addition, only taxa diagnosable by unique combinations of morphological features were recognized as species (Cracraft 1983). Therefore, we recognized species as separately evolving lineages that share a unique combination of morphological characters.

Morphological descriptions follow the general terminology of Lohmann and Taylor (2014). Additional terms follow Radford et al. (1974) for general morphology, Hickey (1973) for leaf shape and venation, Weberling (1992) for inflorescence type, Nogueira et al. (2013) for trichome type, and Gentry and Tomb (1979) or Halbritter et al. (2018) for pollen morphology. All measurements were carried out on dried specimens and/or rehydrated material. In addition, pollen from herbarium specimens were analyzed using scanning electron microscopy (SEM) on a Zeiss DSM 970 scanning electron microscope. Rare character conditions are shown in parentheses in the species descriptions.

Distributions maps were produced in QGIS 2.18 (QGIS Development Team 2018) using a dataset that included a combination of two separate datasets: (i) a newly generated distribution dataset with approximately 150 records from collections made in the field and from digitized specimen labels, and (ii) a dataset with approximately 500 records compiled during the past 25 years (Lohmann unpublished data, described in Meyer et al. 2017).

Preliminary conservation status assessments were performed using the complete distribution dataset using the Geospatial Conservation Assessment Tool (GeoCAT; http://geocat.kew.org/) (Bachman et al. 2011). This tool considers the metrics Extent of Occurrence (EOO) and Area of Occupancy (AOO) for 4 km^2^ grids to objectively estimate conservation status based on the IUCN’s criterion B (IUCN 2012; IUCN Standards and Petitions Subcommittee 2017). We only considered EOO to estimate conservation status of each species as this criterion is less prone to extinction risk overestimation.

The list of examined specimens was produced using the R package monographaR (Reginato 2016).

### Geographic distribution

Members of *Martinella* are distributed in wet forests of Central America, northern South America, the Amazon and the Atlantic Forest of Brazil, between 0–1700 m above sea level (a.s.l.) (Zuntini and Lohmann 2014). Among the five *Martinella* species recognized in this study, *M. obovata* is the most frequent and broadly distributed, occurring from southern Mexico to the southern Amazon, reaching as far south as Bolivia and the Brazilian state of Mato Grosso do Sul. *Martinella insculpta* is also broadly distributed in Central America and Amazonia, but is less common when compared to the sympatric *M. obovata*. The other three species in the genus are rare, with narrow distribution ranges. The only known records of *M. tomentosa* are from Central Brazilian Amazon (Amazonas state), while *M. lanuginosa* is only known from western Brazil (Acre state), Peru, and northern Colombia. Lastly, *M. insignis* is the only member of *Martinella* that is restricted to the Atlantic Forest, occurring in the states of Espírito Santo and Bahia, in eastern Brazil.

### Habitat

Species of *Martinella* occur in wet Neotropical forests. *Martinella insculpta*, *M. lanuginosa*, and *M. tomentosa* predominantly occur in *terra firme* (non-flooded) forests in the Amazon, with some reports of *M. insculpta* growing in white-sand soils. *Martinella obovata* preferentially occurs along riverbanks of the Amazonian white-water rivers, where many individuals were found during field expeditions, and where many collections were made over the years. *Martinella insignis* is restricted to sandy soils of the Brazilian Atlantic Forest, growing close to the shore (Zuntini and Lohmann 2014).

### Reproductive biology

All species of *Martinella* share the flower morphology described by Gentry (1974) as *Martinella*-type flower. This floral morphology is characterized by spathaceous, tubular or urceolate calyces, and by tubular, infundibuliform, or urceolate corollas that are straight and coriaceous, varying in color from red to magenta, or white (Gentry 1974; Alcantara and Lohmann 2010). These traits suggest hummingbird pollination. All Amazonian species of *Martinella* fit the pollination syndrome suggested by their floral morphology, which is supported by field observations, although Euglossini bee visitation was also observed (Gentry 1974). Conversely, *M. insignis* has a yellow corolla that is unique in the genus. This corolla color suggests bee pollination, although field studies are needed to confirm this prediction.

### Economic and ethnobotanical uses

Bignoniaceae species are traditionally used for timber, handicraft, and medication (Gentry 1992). *Martinella* species are broadly used by Amazonian indigenous peoples to treat eye inflammation and conjunctivitis (e.g., Gentry and Cook 1984; Alexiades 1999). Extracts are made using the root’s outer bark, which is scraped, macerated with water, filtered and used as eye drops (e.g., Gentry and Cook 1984; similar reports are available in herbarium specimens’ labels, e.g., G.T. Prance 15557 deposited at INPA and Lewis 14026 deposited at QCNE).

The widespread use of *Martinella* extracts for medicinal purposes throughout the Amazon motivated chemical analyses, which led to the discovery of the alkaloids Martinelline and Martinellic acid from organic extracts of *M. iquitoensis* [= *M. insculpta*] (Witherup et al. 1995). Further studies have been conducted attempting to synthesize these compounds (e.g., Davies et al. 2013).

### Cytology

Chromosome counts for *Martinella obovata* is 2*n* = 40 (Goldblatt and Gentry 1979), which also corresponds to the most common chromosome number in the tribe Bignonieae as a whole (Cordeiro et al. 2017).

### Morphology

**Figure 1.**
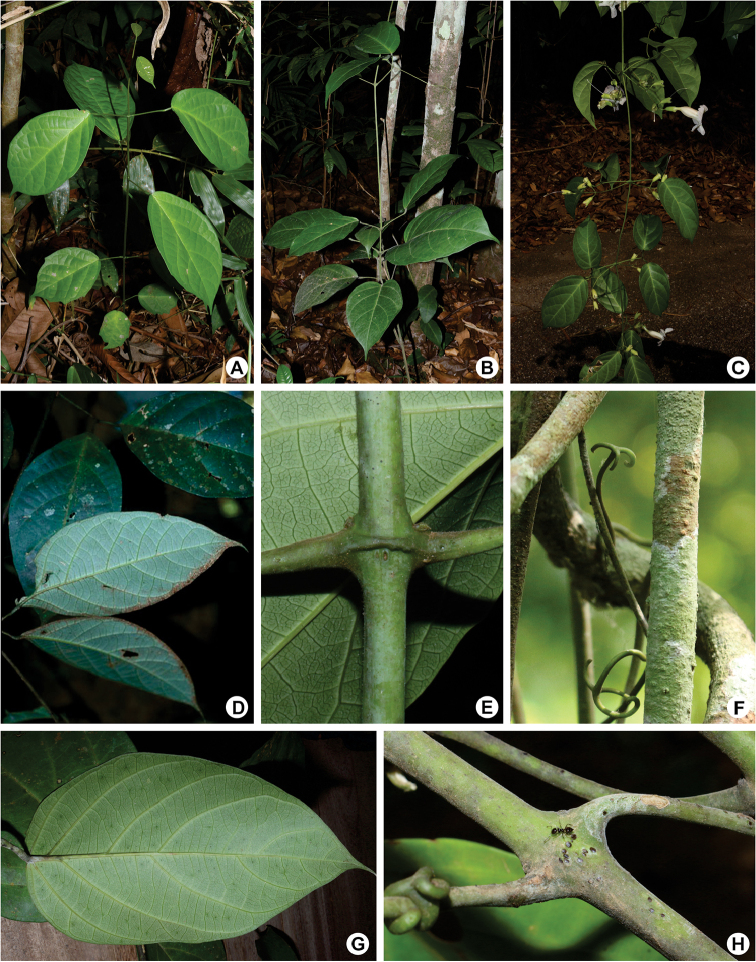
Vegetative characters of *Martinella* Baill **A, B** young individuals of *M. obovata* and *M. insculpta* bearing unifoliolate leaves **C** branch of *M. obovata***D** discolor leaflets of *M. insculpta***E** interpetiolar ridge of *M. obovata***F** trifid tendril of *M. insculpta***G** abaxial side of leaflets of *M. insculpta* with patelliform glandular trichomes **H** interpetiolar region of *M. insculpta* with patelliform glandular trichomes being visited by ants. Photos taken by E.Y. Kataoka, except from photo **D** taken by R. Foster.

*Habit*. All species of *Martinella* are lianas, although seedlings are self-supporting shrubs up to 70 cm tall (see Fig. 1A–C).

*Stems*. Mature stems of *Martinella* bear lenticels and solid pith with four phloem wedges in cross section. Stems with four phloem wedges predominate within tribe Bignonieae and are also found in *Adenocalymma*, *Callichlamys*, *Cuspidaria*, *Fridericia*, *Lundia*, *Manaosella*, *Neojobertia*, *Tanaecium*, *Tynanthus*, *Pachyptera*, *Pleonotoma*, and *Stizophyllum* (Lohmann 2006, Lohmann and Taylor 2014). Young stems are cylindrical and generally remain cylindrical at maturity, becoming tetragonal in *Martinella insculpta*. A continuous ring surrounds the interpetiolar regions of stems (see Fig. 1E). The stem surface is smooth, sparsely to densely covered with trichomes. Stems over ca. 4 cm in diameter often have sparse lenticels. Non-lenticelled parts of the stem are pale to dark green, often with dark blotches.

*Prophylls of the axillary buds*. Structures traditionally referred to as pseudostipules (Gentry 1980) were subsequently shown to represent well-developed prophylls of the axillary buds (Lohmann and Taylor 2014). Prophylls are very useful structures for the identification of genera and species in tribe Bignonieae, often representing morphological synapomorphies of generic-level clades (Lohmann and Taylor 2014). All species of *Martinella* have minute triangular prophylls, a putative morphological synapomorphy of the genus (Lohmann 2006) (see Fig. 1E, H).

*Leaves*. In Bignonieae, leaves are commonly 2-foliolate or 3-foliolate with the terminal leaflet modified in a tendril (Gentry 1980; Lohmann and Taylor 2014). In mature individuals of *Martinella*, leaves are exclusively 2-foliolate, bearing a trifid tendril, with discolor leaflets that bear glandular trichomes on the abaxial side (see Fig. 1C, D, F, G). Young individuals of *M. insculpta* and *M. obovata* show unifoliolate leaves at first and lack tendrils (see Fig. 1A, B). In some taxonomic treatments, *Martinella* was described as bearing simple or trifid tendrils (MacBride 1961; Gentry 1977; Zuntini and Lohmann 2014). However, careful examination of herbarium specimens revealed that most seemingly simple tendrils corresponded to trifid tendrils with missing parts. Trifid tendrils represent the ancestral character state within tribe Bignonieae and were maintained in *Martinella* (Sousa-Baena et al. 2014). Leaf venation is brochidodromous in all species of *Martinella*. Leaf domatia is only found in *M. insignis*, as pocket-like structures at the axil of the midvein with the secondary veins, on the abaxial side of leaflets, mainly at the basal portion.

*Trichomes.* Four main types of trichomes are found in tribe Bignonieae: (i) non-glandular (eglandular) trichomes, (ii) peltate glandular trichomes, (iii) stipitate glandular trichomes, and (iv) patelliform/cupular glandular trichomes (Nogueira et al. 2013). Trichome distribution on the plant body is highly variable, although some genera of tribe Bignonieae are readily recognized by diagnostic patterns of trichome distribution, e.g., *Adenocalymma*, with patelliform glandular trichomes on the prophylls of the axillary buds, floral bracts, calyces, bracteoles, and fruits (Lohmann and Taylor 2014; Fonseca and Lohmann in prep.), and *Pachyptera*, with a field of patelliform glandular trichomes at the interpetiolar region and petiole apex (Francisco and Lohmann 2018). In species of *Martinella*, three trichome types are found: (i) non-glandular trichomes (referred to as simple eglandular trichome hereafter), (ii) stipitate glandular trichomes, and (iii) patelliform glandular trichomes (commonly referred to as “glands” in the literature). The simple eglandular trichomes are distributed throughout the plant, often with density variation in different plant organs; stipitate glandular trichomes are found in high density on the whole plant of *M. insignis* and *M. lanuginosa* and in variable density at different parts of the plant in other species; patelliform glandular trichomes are found on the whole plant of all members of *Martinella*, mainly at the interpetiolar region, and at the base of the abaxial surface of leaflets (see Fig. 1E, H). The patelliform glandular trichomes can also be found on stems, inflorescences, and calyces of *M. insculpta* and *M. obovata*.

**Figure 2.**
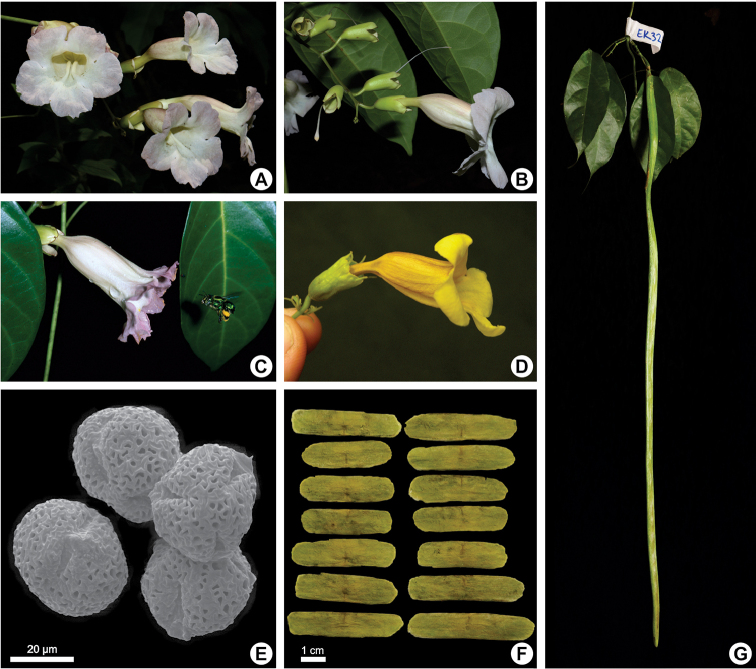
Reproductive characters of *Martinella* Baill **A–C** flowers of *M. obovata* in frontal (**A**) and lateral view (**B, C**) **B** irregularly 3-parted calyces, and corolla narrowly-tubular at base and campanulate at upper portion **C**euglossini bee visiting the flower of *M. obovata***D** lateral view of *M. insignis* flower showing the typical corolla shape and 5-parted calyx **E** scanning electron micrograph showing tricolpate and reticulate pollen grains of *M. obovata***F** dried seeds of *M. obovata* with greenish wings **G** flat, narrow, and long (≤ 1.5 m) fruit of *M. obovata*. All photos taken by E.Y. Kataoka, except from photo **C** taken by G. Gerlach, and **D** taken by A.R. Zuntini.

*Inflorescences.* Flowers of *Martinella* are organized in axillary and/or terminal inflorescences that bear six to 26 flowers, although only a few flowers open at a time. Botryoid inflorescences are found in *M. insculpta*, racemes are found in *M. obovata*, and thyrses are found in *M. insignis*, *M. tomentosa*, and *M. lanuginosa*. Botryoid and racemose inflorescences (see Fig. 2A, B) are lax, contrasting with the congested thyrses, which show higher levels of branching and a higher number of flowers per inflorescence.

*Calyx.* Calyx morphology is highly variable within Bignonieae (Lohmann and Taylor 2014). However, this trait is quite constant within *Martinella*, with the tubular-campanulate and irregularly 2–4-lobed calyx (or regularly 5-lobed in *M. insignis*) representing a diagnostic character (Lohmann 2006; Lohmann and Taylor 2014) (see Fig. 2B–D).

*Corolla.* Corolla shape is an additional distinctive feature in *Martinella*. All species show tubular-campanulate corollas, with a tubular basal portion that is long and much narrower than the markedly campanulate upper portion, giving an inflated appearance compared to the basal portion (see Fig. 2A–D). This distinctive corolla morphology was described by Gentry as the *Martinella*-type flower (Gentry 1974). Corolla color varies from pale lilac (*M. obovata*) to dark magenta (*M. insculpta*) or yellow (*M. insignis*). Corolla color in *Martinella lanuginosa* and *Martinella tomentosa* is unknown, but seems to vary from lilac to magenta, as in other Amazonian species.

*Androecium.* Members of *Martinella* have four didynamous stamens and one very reduced staminode (ca. 1 mm long). The stamens are inserted at the inferior portion of the corolla tube, at approximately 1/4 of the corolla length. Anthers are included, bearing straight and divaricate thecae, and showing glabrous filaments and thecae.

*Pollen.*Gentry and Tomb (1979) highlighted the usefulness of pollen morphology to generic-level identification of members of Bignonieae, although this trait shows convergent evolution. In *Martinella*, pollen is quite constant, tricolpate with reticulate exine (see Fig. 2E) in all species. Similarly, reticulate pollen grains are also found in *Bignonia*, *Mansoa*, *Pachyptera*, and *Pyrostegia* (Gentry and Tomb 1979, Francisco and Lohmann 2018).

*Gynoecium.* As in most Bignonieae, *Martinella* has a bilocular ovary with two fused carpels with axillary placentation (Gentry 1980), a single style with lanceolate and bilamellate stigma. The ovary is terete, with a glabrous and smooth surface.

*Fruits.* Species of *Martinella* have linear, flattened, septicidal capsules with two valves. Capsules of *Martinella* are among the longest fruits in Bignonieae, reaching up to 1.5 m long and 3 cm wide (Fig. 2G), only comparable to *Dolichandra* fruits (Fonseca et al. 2017). In addition, the capsules are glabrous to puberulent.

*Seeds.* Seeds of *Martinella* are symmetrically winged, oblong, and thin (Fig. 2F). The seed body is chartaceous, opaque and brown or green, even when dried (in *M. obovata*). The seed wings are membranous and translucent, brown or green-colored. Like several other clades of Bignoniaceae, seeds are wind-dispersed (Gentry 1979; Gentry 1980).

## Taxonomic treatment

### Martinella

Taxon classificationPlantaeLamialesBignoniaceae

Baill., Hist. Pl. 10: 30. 1888

8D646664-8106-5807-AE4C-E8814251FCF4

#### Type.

*Martinella martini* (DC.) Baill. [= *Martinella obovata* (Kunth) Bureau & K.Schum.].

#### Description.

**Lianas. *Roots*** with swollen portions. ***Branches*** green or light brown, terete, tetragonal, glabrous, puberulous or pubescent, eglandular trichomes simple, glandular trichomes stipitate or patelliform, with a continuous ridge at the interpetiolar region, with few interpetiolar patelliform trichomes; prophylls of the axillary buds minute, glabrous, puberulous or pubescent. ***Leaves*** 2-foliolate with the terminal leaflet generally modified in a trifid tendril; leaflets membranous, chartaceous or coriaceous, glabrous to pubescent, margins entire, revolute, more conspicuously when dried, with or without mite-domatia, with patelliform glands on the adaxial surface. ***Inflorescences*** axillary, botryoid, racemose, or a thyrse. ***Flowers*** with calyces tubular-campanulate, irregularly 2–4(–5)-lobed, lobe apices mucronate or aristate, chartaceous, with scattered patelliform glands; corolla deep purple, lilac, dark magenta or yellow, narrowly tubular at basal portion and wide campanulate at upper portion, straight to slightly curved, membranous, outer surface glabrous, inner surface glabrous with eglandular trichomes concentrated at stamen insertion; stamens included, glabrous, pollen tricolpate and reticulate; ovary terete, smooth, glabrous, with a single series of ovules per placenta, style glabrous, stigma rhombic, glabrous. ***Capsules*** drying brown, linear, flattened, smooth, glabrous, with calyx normally persistent; seeds oblong, winged, with wings opaque, green or beige.

#### Discussion.

The nomenclatural type of *Martinella* has been indicated as *Martinella martinii* (DC.) Baill. ex K.Schum. in earlier treatments of the genus (e.g., Lohmann and Taylor 2014; Zuntini and Lohmann 2014) because Schumann (1894) was the first to explicitly associate the genus name with a species epithet. However, Baillon (1888) provided a footnote that says “Generis typus est *Bignonia Martini* DC.,” representing a valid reference to a previously published basionym, and validly publishing the name *Martinella martini* (DC.) Baill. (see Art. 41.3 and 38.13 of Turland et al. 2018).

As circumscribed here, *Martinella* comprises five species distributed from southern Mexico to eastern Brazil. A key to all species recognized is given below:

### Key to species of *Martinella*

**Table:** 

1	Abaxial surface of leaflets with pocket domatia on the axils of the primary and secondary veins; calyx 5-lobed; corolla yellow; eastern Brazil (Atlantic Forest)	***M. insignis***
–	Abaxial surface of leaflets without pocket domatia; calyx irregularly 2–4-lobed; corolla lilac to deep purple or dark magenta; southern Mexico, the Antilles, Central America and South America (Amazon basin)	**2**
2	Inflorescence racemose	***M. obovata***
–	Inflorescence botryoid or a thyrse	**3**
3	Mature stem quadrangular in cross section; leaflet coriaceous; inflorescence botryoid	***M. insculpta***
–	Mature stem cylindrical in cross section; leaflet chartaceous; inflorescence a thyrse	**4**
4	Branches densely covered with simple eglandular trichomes; leaflets tomentose abaxially	***M. tomentosa***
–	Branches densely covered with stipitate glandular trichomes; leaflets lanuginose abaxially	***M. lanuginosa***

### 1. Martinella insculpta

Taxon classificationPlantaeLamialesBignoniaceae

Sprague & Sandwith, Bull. Misc. Inform. Kew 1934(3): 101. 1934

81E2D7D4-E79E-5009-AB28-4CB35F13A0D0

Fig. 3

Martinella iquitoensis A.Samp., Ann. Acad. Bras. Sci. 7: 123. 1935. [Martinella iquitosensis, orth. var.]. TYPE: PERU. Loreto: Iquitos, 23 Feb 1924, *J.G. Kuhlmann 1492* (holotype: RB-00536899!; isotypes: RB-00537289!, K-000449503 image!, MO-074501 image!).
Martinella manaosiana A.Samp., Bol. Mus. Nac. RJ. 12(3–4): 84. 1936. TYPE: BRAZIL. Amazonas: Manaus, Capuêra de terra firme, Villa Belizario, 25 Jul 1931, *A. Ducke* s.n. [RB 24095] (holotype: RB-00536900!; isotypes: K-000449502 image!, MO-074517 image!, R-000028732!, RB-00537290!).

#### Type.

Guyana. Unknown locality, s.d., *Drake* s.n. (holotype: K-000449501 image!).

**Figure 3.**
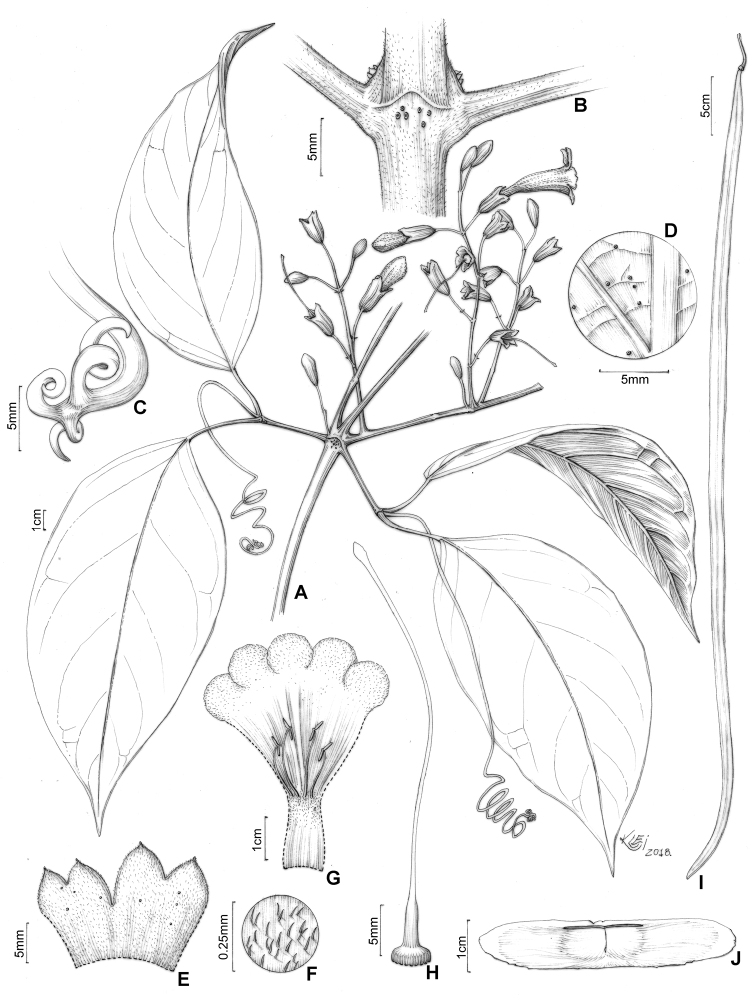
*Martinella insculpta* Sprague & Sandwith **A** flowering branch **B** interpetiolar region with patelliform glandular trichomes and minute and triangular prophylls **C** trifid tendril **D** abaxial side of leaflet with patelliform glandular trichomes **E** calyx external view **F** calyx indumentum **G** open flower showing stamens, trichome distribution, and reduced (ca. 1 mm) staminode **H** gynoecium **I** fruit, flattened with a smooth surface **J** winged seed. Illustrated by Klei Sousa, based on B.L. Stannard 423 (**A, E, G, H**), SPF; E.Y. Kataoka 372 (**B**) and 407 (**C, D**), SPF; G.T. Prance 14914 (**I, J**), INPA; L.O.A. Teixeira 958 (**F**), INPA.

#### Description.

**Lianas**; ***branches*** with solid pith, tetragonal when mature, cylindrical when young, green with dark blotches, drying brown or black, smooth, glabrescent, with simple eglandular trichomes and scattered patelliform glandular trichomes in higher densities at interpetiolar region; prophylls of the axillary buds covered with simple eglandular trichomes, with few patelliform glands. ***Leaves*** 2-foliolate, with the terminal leaﬂet generally modified into a trifid tendril; petioles terete, pulvinated, 31–73 mm long, glabrous, with few patelliform glandular trichomes; petiolules terete, pulvinated, 23–65 mm long, glabrous, with few patelliform glandular trichomes; leaﬂets discolor, with abaxial surface lighter than the adaxial surface, coriaceous, ovate, apex acuminate, base cuneate or truncate, margins entire and slightly revolute, 15–32 × 8.2–23.5 cm, adaxial surface glabrous with simple eglandular trichomes at canaliculi of veins, abaxial surface glabrescent, with patelliform glandular trichomes concentrated near base and scattered along the midvein. ***Inﬂorescences*** botryoid, 8.7–22.3 cm long, puberulent with simple eglandular trichomes, and stipitate and patelliform glandular trichomes; bracts linear, 1.1–1.2 mm long, puberulent, with simple eglandular trichomes and stipitate glandular trichomes; pedicels terete, 11.1–15.2 mm, puberulent, with simple eglandular trichomes and stipitate glandular trichomes. ***Flowers*** with calyx green, chartaceous, campanulate, 11.8–16.8 × 5.5–8 mm, densely covered with simple eglandular trichomes and stipitate glandular trichomes, with few patelliform glandular trichomes, lobes 2–4, apex mucronate, puberulent; corolla dark magenta, membranous, 41.3–69.4 mm long, narrowly tubular basal portion 15–27.4 long × 2.9–4.3 mm wide, upper campanulate portion 26.3–41.9 long × 12–16.6 mm wide, slightly curved, lobes subcircular, ca. 8.7 × 12.7 mm; stamens in two lengths, longer ones 16–21.2 mm, shorter ones 9.8–14 mm, thecae 2.9–3.1 mm, glabrous; staminode ca. 1.8 mm, glabrous; gynoecium 34.8–45.2 mm long; ovary glabrous; style glabrous; stigma lanceolate, glabrous; nectariferous disk ca. 4.3 × 0.9 mm. ***Capsules*** linear, 70–88 × 1.1–1.6 cm, glabrous. *Seeds* ca. 4.7 × 0.9 cm.

#### Distribution and habitat.

**Figure 4.**
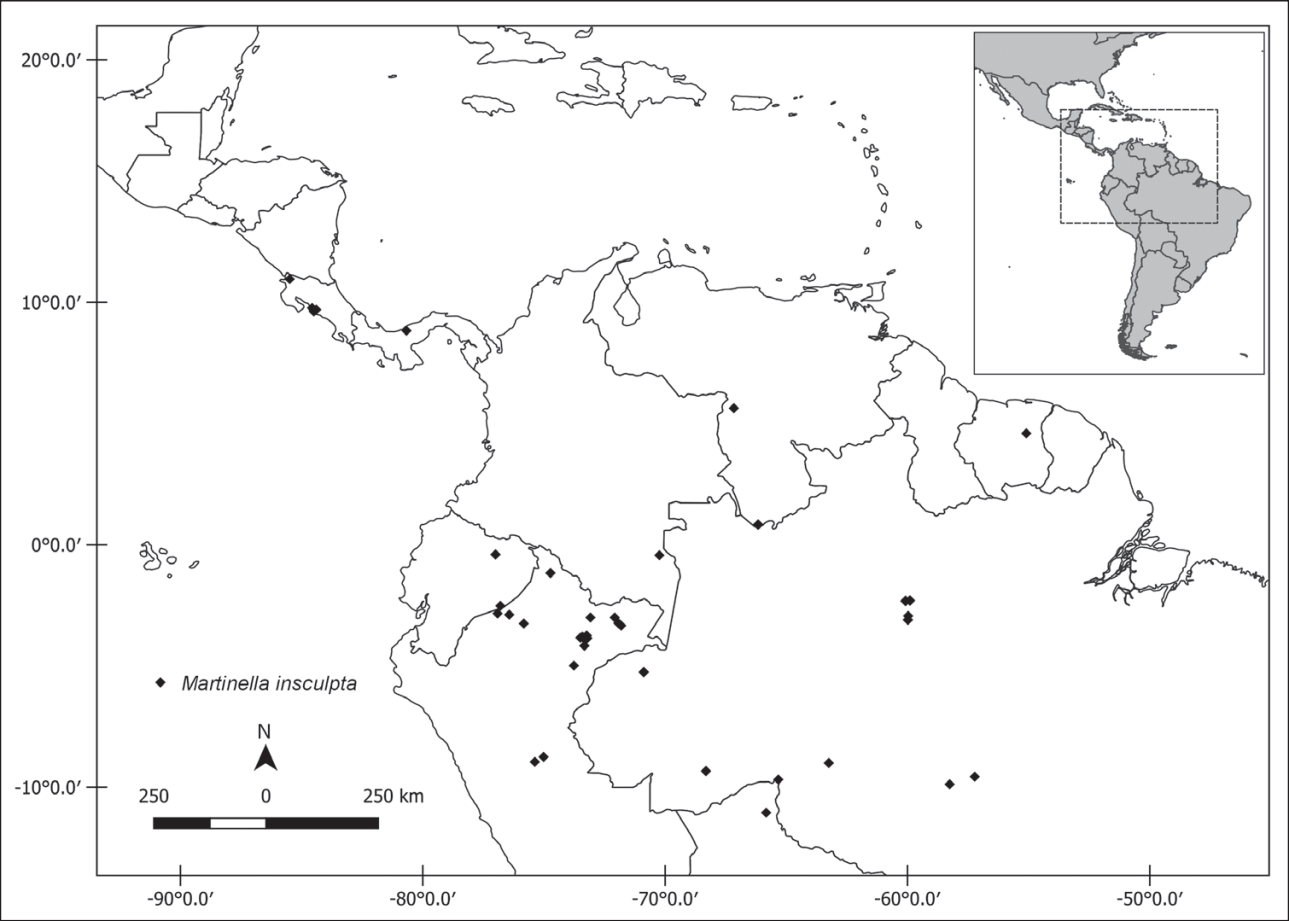
Distribution map of *Martinella insculpta*.

*Martinella insculpta* is widely distributed through Amazonian *terra firme* forests and Central American wet forests (Fig. 4). This species occurs from 59–650 m a.s.l.

#### Etymology.

The specific epithet means carved, engraved, referring to the aspect of leaflet venation on both sides, but especially on the adaxial surface.

#### Phenology.

Flowering specimens were collected from February to November, while fruiting specimens were collected from August to December.

#### Conservation status.

Least Concern (LC) based on the EOO of 4,002,910 km^2^.

#### Discussion.

Here we synonymize *Martinella iquitoensis* under *Martinella insculpta*. Gentry (1973) synonymized *Martinella insculpta* A.Samp. under *M. obovata*, a circumscription that was followed in subsequent taxonomic studies (e.g., Gentry 1977; Gentry 1982). However, the analysis of a large number of living and herbarium specimens of these taxa indicated that the original description and type specimen of *M. insculpta* actually match the circumscription of *M. iquitoensis*. Therefore, *M. iquitoensis* A.Samp. (published in 1935), is here treated as a synonym of *Martinella insculpta* Sprague & Sandwith (published in 1934).

Previous taxonomic studies (Gentry 1982; Zuntini and Lohmann 2014) treated *M. insculpta* and *M. obovata* as part of a species complex due to overlapping morphological characters. However, new collections made in the field and a comprehensive analysis of herbarium specimens collected throughout the known geographic range of this species indicated that these two taxa indeed represent distinct units. Our decision to treat these taxa as separate species was based on the following morphological traits: (i) shape of mature stems in cross section (quadrangular in *M. insculpta* versus cylindrical in *M. obovata*), (ii) apex of petioles and petiolules (pulvinated in *M. insculpta* versus non-pulvinated in *M. obovata*), (iii) leaflet texture and size (coriaceous and consistently larger in *M. insculpta* versus chartaceous and smaller in *M. obovata*), and (iv) inflorescence structure (botryoid in *M. insculpta* and racemose in *M. obovata*). The combination of these distinctive morphological features allows for the correct identification of specimens that are often misidentified in herbarium sheets. In addition, a densely sampled phylogeny of *Martinella* revealed that *M. insculpta* and *M. obovata* represent distinct evolutionary lineages (Kataoka and Lohmann in prep.) providing further support for the recognition of these two taxa as separate species.

#### Common uses.

The bark of the roots of *M. insculpta* and *M. obovata* is used in traditional medicine.

#### Local names.

*Martinella insculpta* and *M. obovata* are known by the popular names *raiz dos olhos* (Brazil), *yuquilla toxju* (Peru), *raíz de ojo*, and *uarakú-manaté* (Venezuela).

#### Specimens examined.

**Bolivia. Beni**: Province of Vaca Diez, 17 km from the road between Riberalta and Guayaramerín on the old road to Cachuela Esperanza, ca. 18 km E of Riberalta, primary forest, 230 m, 11°3'S, 65°50'W, 4 Sept 1981, *J.C. Solomon 6108* (MO). **Brazil. Acre**: Bujari, estrada de acesso à área da Floresta do Antimary sem atividade de manejo florestal, 196 m, 9°19'46.1"S, 68°18'56.2"W, 10 Dec 2016, *E.Y. Kataoka 404* (SPF); Lado direito da entrada da trilha de acesso à torre de observação, 226 m, 9°20'5.3"S, 68°19'13.1"W, 12 Dec 2016, *E.Y. Kataoka 407* (SPF). **Amapá**: Rio Falsino, approx. 10 km upstream of confluence with rio Araguari, west bank, primary forest on terra firme, undulating terrain, 0°50'S, 51°45'W, 29 Sept 1983, *B.V. Rabelo 2386* (SPF). **Amazonas**: Rainforest, vicinity of Maturacá Mission, near rio Maturacá, on trail to heliport, 19 Oct 1970, *J.A. Steyermark 104035* (IAN); Rio Cuieiras just below mouth of rio Branquinho, capoeira, 26 Sept 1971, *G.T. Prance 14914* (INPA, MG, R); Rio Purus, rio Ituxi, serra near Namorado Novo between rio Curuquetê and rio Madeira at Abunã, forest on Terra Firme, 5 Aug 1971, *G.T. Prance 14717* (INPA, MG, R); Barcelos, rio Negro, próximo ao rio Arara, 1 May 1973, *A. Loureiro 37901* (INPA, SPF); Humaitá, Estrada Humaitá-Lábrea, km 59, a 6 km ao norte, mata de terra firme, com muito Babassu, 6 Jun 1982, *L.O.A. Teixeira 958* (INPA, MG); Manaus, 22 Jun 1882, *Schwacke 3610* (RB); A partir da entrada da Reserva pela AM-010 (km 26), estrada sentido alojamento, lado esquerdo na bifurcação entre estrada de acesso ao alojamento e estação meteorológica, 139 m, 2°55'59"S, 59°58'31.9"W, 21 Sept 2016, *E.Y. Kataoka 344* (SPF); Bosque da Ciência do INPA, próximo ao Paiol da Cultura, subindo pela escadaria larga, 84 m, 3°5'56.5"S, 59°59'10.5"W, 20 Sept 2016, *E.Y. Kataoka 342* (SPF); Bosque da Ciência do INPA, próximo ao tanque dos peixes-boi, 69 m, 3°5'50.6"S, 59°59'15.1"W, 20 Sept 2016, *E.Y. Kataoka 339* (SPF); Estrada do Aleixo, km 7, 7 Jul 1977, *W.A. Rodrigues 9707* (INPA, SPF); Estrada do Aleixo, near Manaus, turnoff to rio Negro at km 11 past INPA, 2 Dec 1974, *A.H. Gentry 13024* (INPA); Grounds of INPA at Manaus, 5 Apr 1974, *A.H. Gentry 11208* (INPA); Igapó, 14 Jun 1882, *C.A.W. Schwacke 425* (R); Igapó, 22 Jun 1882, *C.A.W. Schwacke 463* (R); Sede do INPA, estrada do Aleixo, 30 Jul 1973, *P.L. Lisboa 6* (INPA); Presidente Figueiredo, Vila de Balbina, Represa da UHE de Balbina, mata de beira de rio, 27 Jun 2007, *J.A.C. da Silva 1294* (INPA); Rio Preto da Eva, estrada Manaus-Itacoatiara, km 90, rio Preto, terra firme, solo arenoso, 30 Jul 1961, *W. Rodrigues 2203* (INPA); Santa Isabel do Rio Negro, rio Uneiuxi, Makú indian village, 300 km above mouth, indian plantation, 23 Oct 1971, *G.T. Prance 15557* (INPA). **Mato Grosso**: Apiacás, Margem da estrada de acesso Paranaíta – Apiacás, 233 m, 9°33'53.3"S, 57°13'44.5"W, 12 Nov 2016, *E.Y. Kataoka 370* (SPF); Cotriguaçu, Margem da estrada de acesso Nova Bandeirante – Cotriguaçu, ca. 10 Km após travessia do rio Juruena, 251 m, 9°52'40.4"S, 58°15'47.1"W, 13 Nov 2016, *E.Y. Kataoka 372* (SPF). **Rondônia**: Rio Machado, curso inferior, igapó, Feb 1981, *M. Goulding 1324* (MG); Porto Velho, Represa Samuel southern end of E dike near quarry by road, ca. 2 km S of end of main dike, upland hillside forest, 9°00'S, 63°15'W, 7 Jun 1986, *W. Thomas 4974* (INPA). **Colombia. Antioquia**: Primary/old secondary forest on west bank of river, 2 km N of Quebrada La Tirana, tropical Wet/Very Wet Forest Transition zone, rainfall approx. 4400 mm/year, Vic. Planta Providencia 28 km SW of Zaragoza, valley of Río Anorí in areas surrounding the confluence of Quebrada La Tirana and Río Anorí, approx. 3 km upriver from Planta Providencia, 24 Mar 1977, *W.S. Alverson 266* (MO); San Luis, Autopiste Medellín – Bogotá, sector Río Samaná – Río Claro, San Luis – Antioquia, 500 m, 3 Dec 1981, *J.J. Hernandez 99* (QCA). **Costa Rica. Guanacaste**: Liberia, P.N. Guanacaste, cuenca del Tempisque, volcán Orosí, Estación Biológica Maritza, trail to Cacao Station, ca. 1 km from Maritza comedor, ~ 100 km into first patch of primary forest, N side of trail, 0.1 ha Transect Maritza, premontane moist forest, 650 m, 10°57'19.1748"N, 85°29'29.6432"W, 12 Mar 2003, *B. Boyle 7052* (MO). **Puntarenas**: Cantón de Garabito, R.B. Carara, cuenca del Tárcoles, sector Bijugual, entrando a los bosques poco intervenidos, 600 m, 9°46'0"N, 84°34'0"W, 14 May 1998, *A. Rodríguez 3367* (MO). **San José**: Turrubares, San Juan De Mata, area no protegida, Montelimar, 85 m, 9°37'24"N, 84°29'55"W, 15 Oct 2001, *A. Estrada 3072* (MO); Z.P. La Cangreja, Mastatal de Puriscal, bosque primario en parches remanentes, 300 m, 9°41'45"N, 84°23'47"W, 21 Oct 1992, *J.F. Morales 910* (MO). **Ecuador. Napo**: Coca (Puerto Francisco de Orellana), 8 km al N de Coca, bosque humedo tropical, suelo aluvial fertil, bosque secundario, 250 m, 0°24'S, 77°0'W, 8 Apr 1985, *W. Palacios 272* (MO, QCA, QCNE); **Pastaza**: Kapawi, Río Pastaza, village area, secondary and primary forests, and pastures, 235 m, 2°31'S, 76°48'W, 25–29 Jul 1989, *W.H. Lewis 14026* (QCNE). **Panama.** Colón, Teck Cominco Petaquilla mining concession, forest along road, 220 m, 8°49'39"N, 80°40'28"W, 20 Feb 2008, *G. McPherson 20083* (MO). **Peru. Huanuco**: Carretera marginal (in construction) km 4–12 south from Km. 86 of Pulcallpa-Tingo Maria road, 270 m, 8°45'S, 75°1'W, 1 Jun 1983, *A.H. Gentry 41384* (MO). **Loreto**: Pampa hermosa and vicinity, Río Corrientes, 1 km S of junction with Río Macusari, low rainforest, mostly terra firma with scattered white sand, 160 m, 3°15'S, 75°50'W, 3–20 Dec 1985, *W.H. Lewis 9975* (MO); Alto Amazonas, Puranchim, río Sinchiyacu, rainforest, terra firma and palm lowlands, 200 m, 2°50'S, 76°55'W, 3–7 Dec 1988, *W.H. Lewis 14389* (MO); Alto Amazonas, Andoas, Capihuari, 5 km NE of Andoas on Río Capihuari, near Ecuador border along oil pipeline, lateritic uplands alternating with Mauritia swamps, 240 m, 17 Nov 1979, *A.H. Gentry 28200* (MO); Primary forest behind Brillo Nuevo, Pebas, Río Yaguasyacu, Brillo Nuevo, 11 Sept 1981, *R. Hahn 123* (MO). **Maynas**: Iquitos, Carretera de Peña Negra, ca. 7 km de Quisto Cocha, en terreno arenoso, monte despejado, 150 m, 12 Jul 1982, *M. Rimachi Y. 6180* (MO); Carretera Iquitos-Nauta, km 45, bosque primario, 120 m, 4°10'S, 73°20'W, 12 Jun 1987, *R. Vásquez 9172* (MO); Iquitos, trail between extension of Yavari and Versailles, mostly highly disturbed upland scrub area, 11 Feb 1974, *M. Rimachi Y. 835* (MO); Puerto Almendras, Río Nanay, bosque primario en suelo con arena blanca, 122 m, 3°48'S, 73°25'W, 18 Jul 1988, *R. Vásquez 10977* (MO); Río Nanay, Carretera de Picuruyacu, trocha de la granja de la marina, en terreno arenoso, 160 m, 26 Jul 1982, *M. Rimachi Y. 6270* (MO); Río Momon, Momoncillo (Caserio), borde de pastizal de ganado, 17 Aug 1976, *J. Revilla 979* (MO); Santo Tomas (Iquitos), 100 m, 3°51'S, 73°13'W, 14 Apr 1979, *F. Ayala 1793* (MO); Napo, Environs of Río Santa María, collected one hour upstream of the Secoya village of “Vencedor”, 4 hours by outboard from the mouth of the Santa Maria river, 100 m, 1°10'S, 74°44'W, 15 May 1982, *S.R. King 492* (MO). **Suriname. Brokopondo**: Brownsweg, near Brownsweg Nature Park, little disturbed high mesophytic rain forest on slope, gravelly clay soil, 240 m, 4°35'41.28"N, 55°6'0"W, 1 Oct 2005, *K. Van Kerckhove MVK 114* (SPF). **Venezuela. Amazonas**: Atures, transecto desde bosque alto denso con tatucos a orillas de río Cataniapo, hasta bosque medio ralo en parte alta de colina, a 1 km al oeste de San Pedro de Cataniapo, a unos 60 km al sur-este de Puerto Ayacucho, 100 m, 5°38'N, 67°10'W, 7 Mar 1981, *F. Guanchez 923* (MO); Río Negro, 0 to 2 km west of Cerro de La Neblina base camp, which is on Río Mawarinuma, 140 m, 0°50'N, 66°10'W, 7 Feb 1984, *R. Liesner 15711* (MO); Near Cerro de La Neblina base camp, which is on Río Mawarinuma, 140 m, 0°50'N, 66°10'W, 25 Mar 1984, *R. Liesner 16956* (MO); Río Negro, Neblina Massif, bongo (dugout) trip down rio Mawarinuma for c. 2 km + NW from base camp at mouth of canyon, 140 m, 0°50'N, 66°10'W, 31 Mar 1984, *B.L. Stannard 423* (SPF).

### 2. Martinella insignis

Taxon classificationPlantaeLamialesBignoniaceae

A.H.Gentry ex Zuntini & L.G.Lohmann, Phytokeys 37: 17–21. 2014

A6961E9D-B007-5302-A824-79D5742C3EB6

Fig. 5

#### Type.

Brazil. Bahia: Itamaraju, Rodovia Itamarajú-Teixeira de Freitas, 3 km de Itamarajú (BR-101). Fazenda Chapadão, 3 Nov 1983, *R. Callejas*, *A.M. de Carvalho & L.M. Silva 1629* (holotype: MBM-94960 image!; isotypes: K-000977667 image!, MO-074484 image!, NY-00483568 image!, RB-00058792!, CEPEC-00034725 not seen).

**Figure 5.**
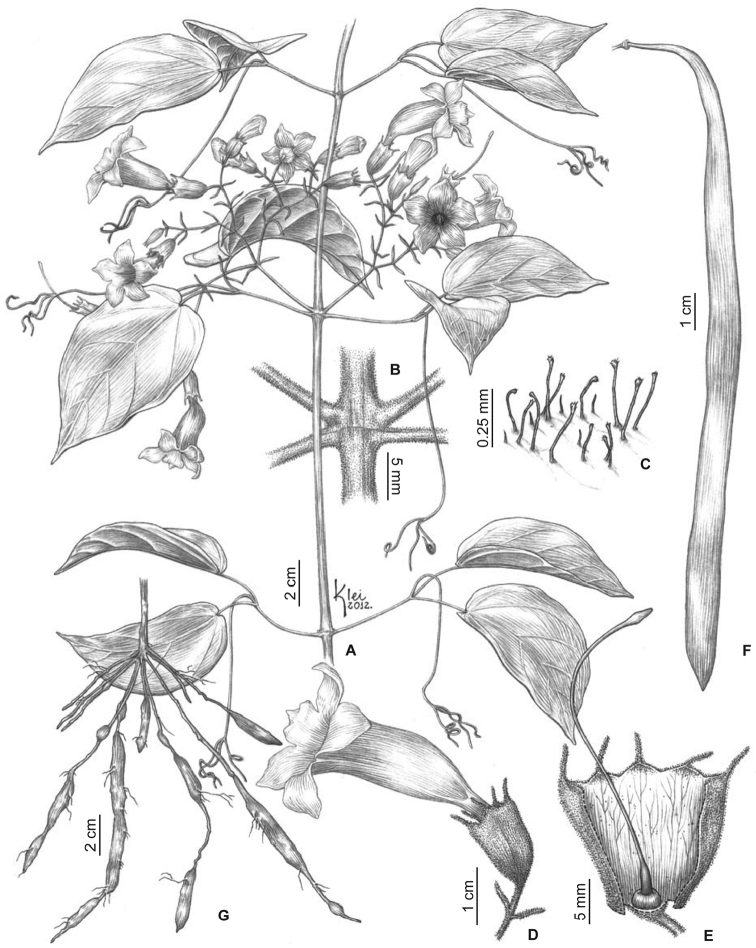
*Martinella insignis* A.H. Gentry ex Zuntini & L.G. Lohmann **A** flowering branch **B** interpetiolar region **C** stipitate glandular trichomes **D** flower in lateral view **E** calyx (opened) and gynoecium **F** fruit **G** roots with swollen portions. Illustration reproduced from Zuntini and Lohmann (2014); illustrated by Klei Sousa, based on *A.R. Zuntini 151* (**A–E**) and *321* (**G**), SPF; *D. Sucre 5519* (**F**), RB.

#### Description.

**Lianas**; ***branches*** with solid pith, cylindrical, green, drying brown, striated, pubescent, densely covered with stipitate glandular trichomes; prophylls of the axillary buds densely covered with stipitate glandular trichomes. ***Leaves*** 2-foliolate, with the terminal leaﬂet generally modified into a trifid tendril; petioles terete, not pulvinated, 34.6–48.4 mm long, covered with stipitate glandular trichomes; petiolules terete, not pulvinated, 13.9–26.4 mm long, covered with stipitate glandular trichomes; leaﬂets discolor, with abaxial surface lighter than the adaxial surface, membranous, ovate, apex acuminate to caudate, base cordate, margins entire and slightly revolute, 6.4–8.8 × 3.6–5.2 cm, adaxial surface glabrous, with stipitate glandular trichomes on the margins and at the canaliculi of main veins, abaxial surface pubescent, densely covered with stipitate glandular trichomes at main veins, pocket domatia on the axils of primary and secondary veins, few patelliform glandular trichomes concentrated near base and scattered along the midvein. ***Inﬂorescences*** in compound thyrses, 7.5–11.5 cm long, sparsely to densely covered with stipitate glandular trichomes; bracts linear to narrowly elliptic, 5.2–20.2 × 0.6–2.5 mm, pubescent, densely covered with stipitate glandular trichomes; pedicels terete, 6.3–11.2 mm, sparsely to densely covered with stipitate glandular trichomes. ***Flowers*** with calyx pale green, chartaceous, campanulate, 13.1–17.2 × 6.1–11.9 mm, densely covered with stipitate glandular trichomes, lobes 5, apex aristate, aristae 1.8–4.3 mm long, pubescent, densely covered with stipitate glandular trichomes; corolla yellow, membranous, 41.5–47.3 mm long, narrowly tubular basal portion 15.8–18.5 mm long × 2.5–4.9 mm wide, upper campanulate portion 23.1–32 mm long × 12.5–15.2 mm wide, slightly curved, lobes subcircular, 7.5–9.1 × 8.6–9.5 mm; stamens in two lengths, longer ones 12.4–13.3 mm, shorter ones 12.0–12.5 mm, thecae 2.5–2.7 mm, glabrous; staminode 1.3–3.4 mm, glabrous; gynoecium 30.9–35 mm long; ovary glabrous; style glabrous; stigma lanceolate, glabrous; nectariferous disk 2.3–3.1 × 1.0–1.2 mm. ***Capsules*** linear, 40.2–90 × 1.1–1.5 cm, pubescent when immature, glabrous when developed. ***Seeds*** ca. 4.5 × 1.2 cm.

#### Distribution and habitat.

**Figure 6.**
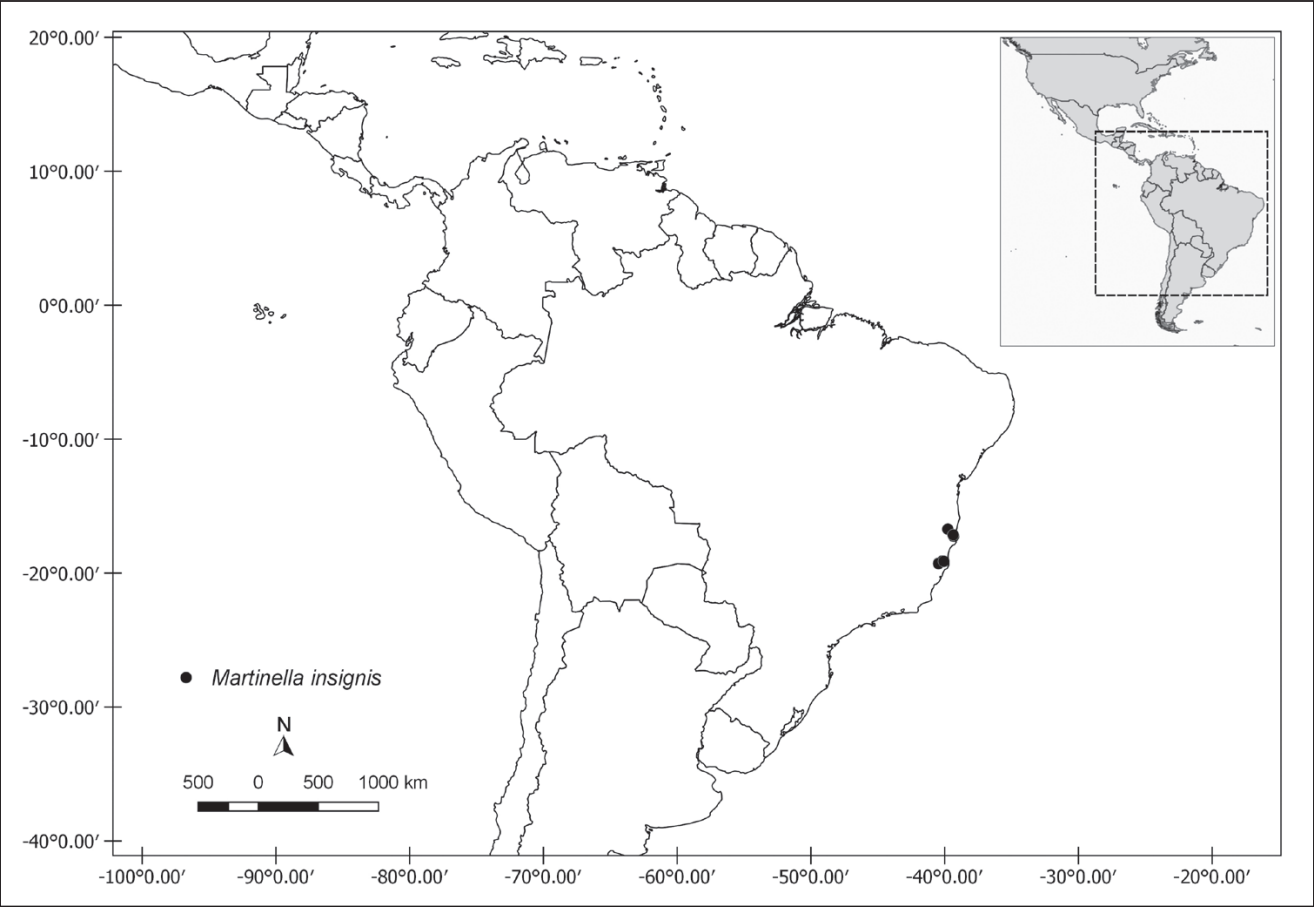
Distribution map of *Martinella insignis*.

*Martinella insignis* is endemic to the Atlantic Forest of Eastern Brazil (see Fig. 6), where it grows in sandy soils, in areas between 62–590 m a.s.l.

#### Etymology.

The specific epithet means conspicuous, readily distinguishable, referring to the contrasting corolla color when compared to other species.

#### Phenology.

Flowering specimens were collected between October and February, while fruiting specimens were collected in January and November.

#### Conservation status.

Vulnerable (VU) based on the EOO of 12,721 km^2^.

#### Discussion.

*Martinella insignis* is the only representative of the genus that occurs in the Atlantic Forest, where it is rare, with only a few herbarium collections available to date. In addition, *M. insignis* is remarkably distinctive from its congeneric species due to the membranous leaflets with pocket-shaped domatia on the abaxial surface, 5-lobed and aristate calyces, and yellow corollas.

#### Specimens examined.

**Brazil. Bahia**: RPPN Fazenda Riacho das Pedras, prop. Gersino Antônio Bronzon, Mata de Tabuleiro com pequenos distúrbios (extração seletiva), 78 m, 17°8'48"S, 39°21'53"W, 12 Feb 2007, *R.A.X. Borges 825* (RB, SPF). **Espírito Santo**: Governador Lindemberg, Pedra de Santa Luzia, prop. Firmino Sottele, 420–590 m, 19°17'17"S, 40°27'56"W, 7 Nov 2007, *V. Demuner 4481* (SPF); Linhares, Reserva Natural Vale. MME, 5 Oct 2011, *A.R. Zuntini 321* (SPF); Sooretama, Reserva Natural da Companhia Vale do Rio Doce (“Reserva de Linhares”), 62 m, 19°6'59.7"S, 40°4'21.7"W, 14 Dec 2007, *A.R. Zuntini 151* (SPF).

### 3. Martinella lanuginosa

Taxon classificationPlantaeLamialesBignoniaceae

Kataoka & L.G.Lohmann sp. nov.

45968FDF-FB89-5ADC-ADE5-3928CD8BC12F

urn:lsid:ipni.org:names:77217116-1

Fig. 7

#### Type.

Peru. Madre de Dios: Tambopata, Dist. Puerto Maldonado, Fundo Concepción, bosque ribereño, 200 m, 12°32'S, 69°03'W, 22 Aug 2003, *I. Huamantupa*, *J. Vargas & J. Quispe*, *3698* (holotype: SPF-240817!; isotypes: MO-2981780 not seen, AMAZ not seen, CUZ not seen, HUT not seen, USM not seen).

#### Diagnosis.

*Martinella lanuginosa* differs from other Amazonian species of *Martinella* by the lanuginose leaflets on the abaxial surface and inflorescences arranged in lax thyrses, contrasting with the glabrous or tomentose leaflets on the abaxial surface and inflorescences botryoid or racemose found in all other Amazonian species.

**Figure 7.**
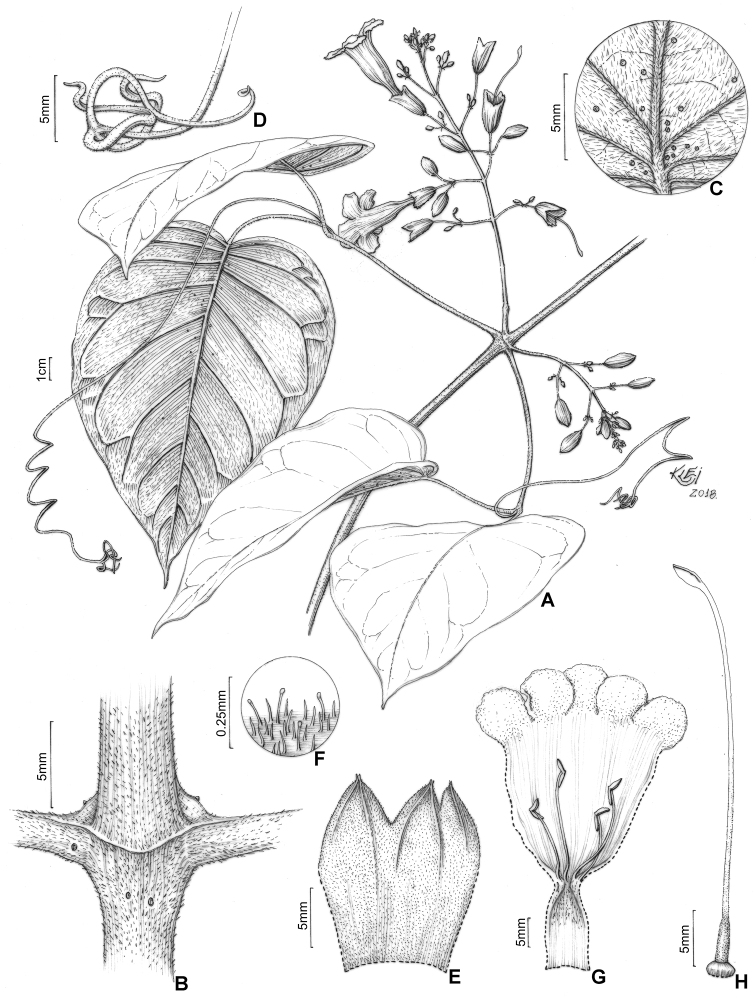
*Martinella lanuginosa* Kataoka & L.G. Lohmann **A** flowering branch **B** interpetiolar region with patelliform glandular trichomes **C** abaxial side of leaflet with lanuginose indumentum and patelliform glandular trichomes **D** trifid tendril **E** calyx external view **F** detail of calyx indumentum **G** open flower showing anthers, trichome distribution, and reduced (ca. 1.5 mm) staminode **H** gynoecium. Illustrated by Klei Sousa, based on *A.H. Gentry 27233* (**B, D**), MO; *I. Huamantupa 3698* (**A, E, F–H**), *R. Rueda 414* (**C**), SPF.

**Figure 8.**
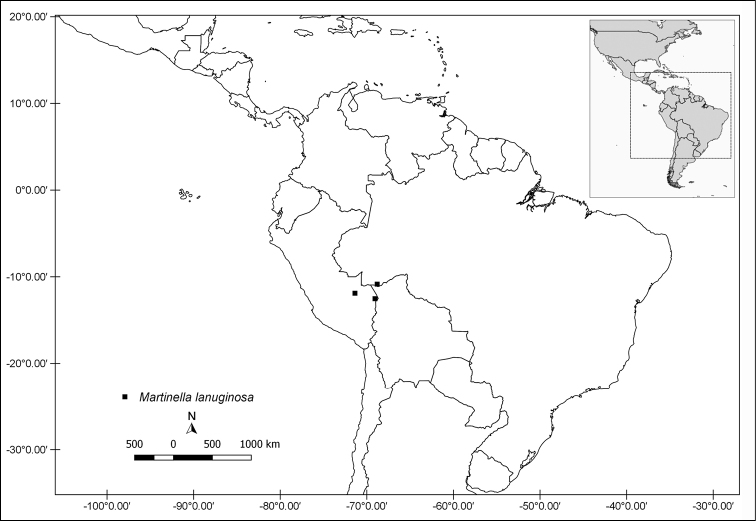
Distribution map of *Martinella lanuginosa*.

#### Description.

**Lianas**; ***branches*** with solid pith, cylindrical, green, drying light brown, smooth, pubescent, densely covered with stipitate glandular trichomes, with scattered patelliform glandular trichomes more frequently at interpetiolar region; prophylls of the axillary buds densely covered with stipitate glandular trichomes. ***Leaves*** 2-foliolate with the terminal leaﬂet generally modified into a trifid tendril; petioles terete, not pulvinated, 40–83.7 mm long, densely covered with stipitate glandular trichomes with few scattered patelliform glandular trichomes; petiolules terete, not pulvinated, 19.4–70.1 mm long, densely covered with stipitate glandular trichomes and occasional patelliform glandular trichomes; leaﬂets discolor, with abaxial surface lighter than the adaxial surface, chartaceous, ovate, apex acuminate, base cordate, margins entire and slightly revolute, 6.5–14.4 × 5.2–11.2 cm, adaxial surface glabrous, with simple eglandular trichomes and stipitate glandular trichomes at canaliculi of veins, abaxial surface lanuginose, densely covered with simple eglandular trichomes with few patelliform glandular trichomes distributed along the midvein. ***Inﬂorescences*** in thyrses, 6.5–11 cm long, densely covered with simple eglandular trichomes and stipitate glandular trichomes, with few patelliform glandular trichomes; bracts linear, 1.5–3 mm long, puberulent, densely covered with stipitate glandular trichomes; pedicels terete, 6.5–15.5 mm, densely covered with stipitate glandular trichomes. ***Flowers*** with calyx chartaceous, campanulate, 18–21.6 × 8.9–12.3 mm, puberulent, covered with simple eglandular trichomes and stipitate glandular trichomes, lobes 2–4, apex mucronate, puberulent; corolla lilac, membranous, 62.6–69.6 mm long, narrowly tubular basal portion 20.6–21.9 mm long × 5.3–6.2 mm wide, upper campanulate portion 42–47.7 mm long × 16.9–20.7 mm wide, lobes subcircular, 7.3–8.3 × 12.4–13 mm; stamens in two lengths, longer ones 17.9–18.6 mm, shorter ones 12.3–13.3 mm, thecae 3–3.2 mm, glabrous; staminode ca. 1.2 mm, glabrous; gynoecium ca. 42 mm long; ovary glabrous; style glabrous; stigma lanceolate, glabrous; nectariferous disk 3.3–3.4 × 1.1–1.15 mm. ***Fruits and seeds*** not seen.

#### Distribution and habitat.

*Martinella lanuginosa* is restricted to *terra firme* (non-flooded) forests of western Amazon (see Fig. 8), with known occurrences in Brazil (Acre state) and eastern Peru.

#### Etymology.

The specific epithet relates to the lanuginose indumentum on the abaxial surface of leaflets that confers a wool-like feel when touched.

#### Phenology.

Flowering specimens were collected in late October.

#### Conservation status.

Near Threatened (NT) based on the EOO of 24,543 km^2^.

#### Discussion.

*Martinella lanuginosa* is a new taxon discovered based on morphology and confirmed to represent an independent lineage based on molecular phylogenetic data (Kataoka and Lohmann in prep.). This new taxon is sister to *M. insculpta* and *M. obovata*. *Martinella lanuginosa* is readily distinguished from its Amazonian sister-taxa by the leaflets lanuginose on the abaxial side (versus glabrescent in *M. insculpta*), and glabrous in *M. obovata* (versus tomentose in *M. tomentosa*). The inflorescence is a lax thyrse that is easily distinguished from the densely arranged thyrse of *Martinella tomentosa*. Very few specimens of *M. lanuginosa* have been collected, none of which bear fruits and/or seeds.

#### Specimens examined.

**Brazil. Acre**: Brasileia, Reserva Extrativista Chico Mendes, seringal Porongaba, colocação Dois Irmãos, terra firme, 10°51'N, 68°48'W, 28 Oct 1991, *L. Ferreira 109* (NY). **Colombia**. **Bolívar**: Turbaco, Fundación Jardín Botánico “Guillermo Piñeres” y alrededore, 130 m, 23 May 1992, *R. Rueda 414* (MO). **Peru**. **Loreto**: Alto Amazonas, Yurimaguas Estación Experimental de North Carolina State, 10 years old second growth dominated by *Cecropia*, on sandy and yellow lateritic soil, 180 m, 9 Oct 1985, *A.H. Gentry 52144* (MO). **Madre de Dios**: Cocha Cashu camp, Manú National Park, Río Manú, mature forest on alluvial soil, 380 m, 24 Oct 1979, *A.H. Gentry 27233* (MO); Manú, Parque Nacional Manú, Estación Biologica de Cocha Cashu. Mature floodplain, 150 m, 11°54'S, 71°22'W, 6 Jun 2001, *L.G.Lohmann 616* (MO).

### 4. Martinella obovata

Taxon classificationPlantaeLamialesBignoniaceae

(Kunth) Bureau & K.Schum., in Mart., Fl. Bras. 8(2):161, tab. 84. 1896

AA9F6507-3CFB-5D89-84C1-E2B8E94D2501

Fig. 9

Spathodea obovata Kunth, Nov. Gen. Sp. (quarto ed.) 3: 147. 1819 [1818]. Bignonia obovata (Kunth) Spreng., Syst. Veg. 2:830. 1825. Macfadyena obovata (Kunth) Miers, Proc. Roy. Hort. Soc. London 3: 200. 1863. TYPE. Colombia. Magdalena: Turbaco, s.d., *F.W.H.A. von Humboldt & A.J.A. Bonpland 1391* (holotype: P-00670823 image!).
Bignonia fockeana Miq., Linnaea 18: 609. 1844. Macfadyena fockeana (Miq.) Miers, Proc. Roy. Hort. Soc. 3: 200. 1863. TYPE. Suriname. Paramaribo, Aug 1844, *H.C. Focke 924* (lectotype, designated by Gentry 1974 [1973], p. 877: U-0000750 image!).
Tabebuia cordata Benth., Bot. Voy. Sulphur 129. 1845 [1844]. TYPE. Panama. Isthmus of Darién, s.d., G.W. *Barclay s.n.* (lectotype, designated by Gentry 1974 [1973]: K-000449504 image!).
Bignonia martini DC., Prodr. 9: 152. 1845. [as Martini]. Martinella martini (DC.) Baill. ex K.Schum. In Engler & Prantl, Nat. Pflanzenf. 4(3b): 216. 1894. TYPE. French Guiana. Cayenne, s.d., J. *Martin s.n.* (lectotype, first-step designated by Gentry 1974 [1973], p. 877 [as “type”], second-step designated here: P-00481520 image!; isolectotypes: P-00481521 image!, P-00481522 image!, U-0000749 image!, US-00125833 image!).
Doxantha longisiliqua Miers, Proc. Roy. Hort. Soc. 3: 190. 1863. Bignonia longisiliqua Bertero ex Spreng., Syst. Veg. 2: 830. 1825, non Jacq. 1780 nec Vell. 1829. TYPE: Colombia. S. Martha, s.d., C.L.G. *Bertero s.n.* (neotype, designated here: G-DC-00133287 image!).
Martinella gollmeri K.Schum. Engler & Prantl, Nat. Pflanzenf. 4(3b): 216. 1894. TYPE: Venezuela, s.d., J. Gollmer s.n. (B probably destroyed); Amazonas, road from San Fernando de Atabapo to Santa Barbara, 12–40 Km from San Fernando, 24 Mar 1974, A.H. Gentry & S.S. Tillet 10864 (neotype, designated here: MO-074427!).
Anemopaegma leptosiphon Rusby, Mem. New York Bot. Gard. 7: 354. 1927. TYPE. Bolivia. Ixiamos, 245 m, 15 Dec 1921, *M. Cardenas 1926* (holotype: NY-00313067 image!; isotype: BKL-00002909 image!).
Arrabidaea duckei A.Samp., Bol. Mus. Nac. Rio de Janeiro 12(3–4): 81. 1936. Periarrabidaea duckei (A.Samp.) A.Samp., Ann. Acad. Bras. Sci. 12: 91. 1936. TYPE. Brazil. Manaus, s.d., A. *Ducke s.n.* (holotype [two sheets]: sheet 1, RB-00536852!, sheet 2, RB-00536853!; isotype: R-28626 [accession number]!).

#### Description.

**Lianas**; ***branches*** with solid pith, cylindrical, green, drying brown, smooth, puberulent, covered with few stipitate glandular trichomes, with scattered patelliform glandular trichomes more frequently at interpetiolar region; prophylls of the axillary buds covered with simple eglandular trichomes. ***Leaves*** 2-foliolate, with the terminal leaﬂet generally modified into a trifid tendril; petioles terete, not pulvinated, 8.5–66 mm long, covered with stipitate glandular trichomes, with few scattered patelliform glandular trichomes; petiolules terete, not pulvinated, 5–40.4 mm long, covered with stipitate glandular trichomes, with occasional patelliform glandular trichomes; leaﬂets discolor, with abaxial surface lighter than the adaxial surface (silver-like color), chartaceous, ovate, apex acuminate, base cordate, margins entire and slightly revolute, 7.9–13.2 × 3.0–9.4 cm, adaxial surface glabrous, with simple eglandular trichomes and stipitate glandular trichomes at canaliculi of veins, abaxial surface glabrescent, with patelliform glandular trichomes concentrated near the base and scattered along the midvein. ***Inﬂorescences*** racemose, 11.9–15.5 cm long, sparsely covered with simple eglandular trichomes and stipitate and patelliform glandular trichomes; bracts linear, 1.5–2 mm long, puberulent, densely covered with simple eglandular trichomes and stipitate glandular trichomes; pedicels terete, 4–12.7 mm, puberulent, with simple eglandular trichomes and stipitate glandular trichomes. ***Flowers*** with calyx green, chartaceous, campanulate, 11.9–17.6 × 4.8–9.6 mm, puberulent, sparsely covered with simple eglandular trichomes and stipitate glandular trichomes, with few patelliform glandular trichomes, lobes 2–4, apex mucronate, puberulent; corolla light lilac to dark magenta, membranous, 55.2–58.5 mm long, narrowly tubular basal portion 15.6–17.2 long × 4.2–5.3 mm wide, upper campanulate portion 16.1–18.7 mm long × 14–15 mm wide, lobes subcircular, 10.5–11.7 × 12.7–15.7 mm; stamens in two lengths, longer ones 16–20 mm, shorter ones 12–12.6 mm, thecae 2.5–3.1 mm, glabrous; staminode 0.8–1 mm, glabrous; gynoecium 34–36.3 mm long; ovary glabrous; style glabrous; stigma lanceolate, glabrous; nectariferous disk 3.2–3.6 × 1.3 mm. ***Capsules*** linear, 20.5–116 × 1.1–2 cm, glabrous. ***Seeds*** ca. 4 × 1.1 cm.

**Figure 9.**
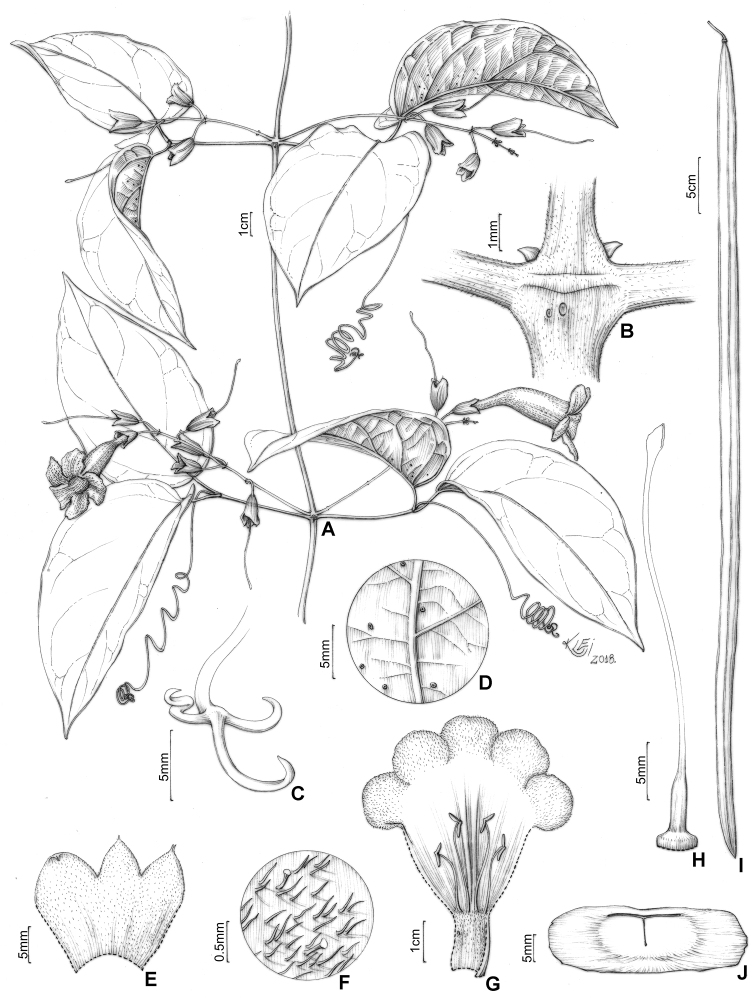
*Martinella obovata* (Kunth) Bureau & K. Schum **A** flowering branch **B** interpetiolar region with minute and triangular prophylls **C** trifid tendril **D** abaxial side of leaflet with patelliform glandular trichomes **E** calyx external view **F** detail of calyx indumentum **G** open flower showing anthers, trichome distribution, and reduced (ca. 1 mm) staminode **H** gynoecium **I** fruit flattened with a smooth surface **J** winged seed. Illustrated by Klei Sousa, based on *E.Y. Kataoka 309* (B), *329* (**C, J**) and *360* (**A, D–I**), SPF.

#### Distribution and habitat.

**Figure 10.**
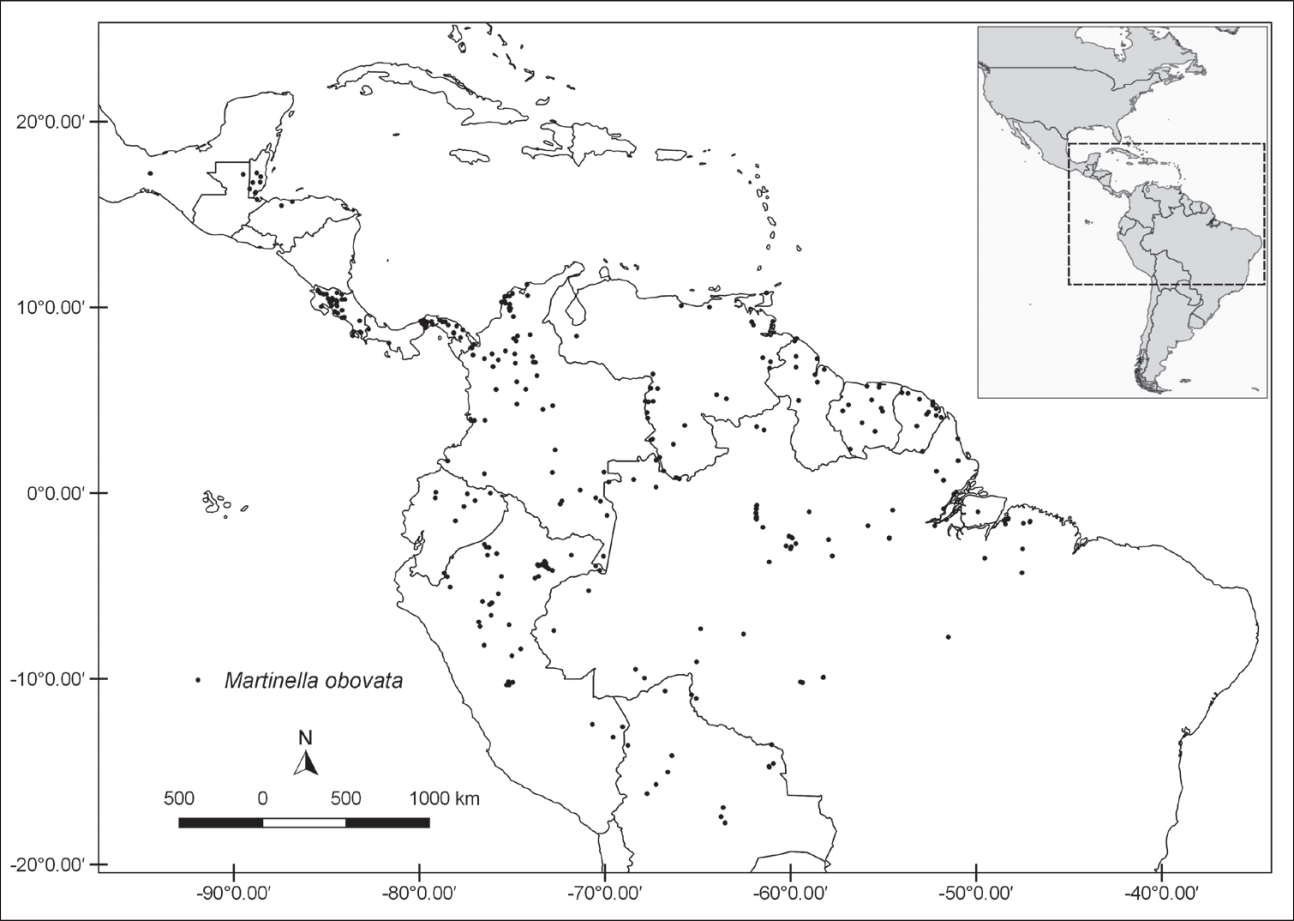
Distribution map of *Martinella obovata*.

*Martinella obovata* is widely distributed through Amazonian *terra firme* forests and wet forests of southern Mexico and Central America (Fig. 10). This species occurs between 0–1700 m a.s.l., in a wide range of forested habitat types.

#### Phenology.

Flowering and fruiting specimens were collected throughout the year.

#### Conservation status.

Least Concern (LC) based on the EOO of 10,477,635 km^2^.

#### Discussion.

*Martinella obovata* is diagnosable by a combination of cylindrical stems in cross section, glabrous leaflets, racemose inflorescence, and calyx covered with simple eglandular and stipitate glandular trichomes. *Martinella obovata* is the most widely distributed species in the genus and occurs from southern Mexico to the southern Amazon in a wide altitudinal range. Despite the wide geographic and elevational range, no clear morphological discontinuities were identified in our study. However, as expected, we did find some variation especially considering plasticity to local environmental conditions, such as thicker leaves in specimens collected at localities associated with rocky outcrops. Based on the morphological evidence and robust molecular phylogenetic data (Kataoka and Lohmann in prep.), we recognize a broadly distributed *M. obovata*.

The widespread *M. obovata* recognized here includes multiple synonyms, several of which required the designation of lectotypes or neotypes. During Alwyn Gentry’s prolific career, he published many taxonomic treatments and Floras in which he listed several types and unintentionally designated lectotypes. For example, Gentry (1973) unintentionally designated lectotypes for *Bignonia fockeana* Miq. and *Tabebuia cordata* Benth. The specimens designated as lectotypes are in good condition, allowing for an accurate identification of these taxa, representing good lectotypes.

Some of Gentry’s unintentional lectotypes were first-step lectotypifications (see Art. 9.17 of Turland et al. 2018), requiring second-step lectotypes. When appropriate, the required second-step lectotypes are proposed here. For example, in the protologue of *Bignonia martini*, De Candolle (1845) specifically cited a specimen collected by Martin in Cayenne (French Guiana). Gentry (1973) cited as “type” collections made by J. Martin in French Guiana deposited at U and US. We found additional collections made by J. Martin in Cayenne that were annotated as “Type” and “Isotype” deposited at P. One of those specimens bears flowers, is in good condition and has a stamp that indicates “Type”. This material is here selected as a second-step lectotype. Duplicates (isolectotypes) of the type collection are deposited at P, U and US.

*Bignonia longisiliqua* Bertero ex Spreng. (Sprengel 1825) and *Bignonia longisiliqua* Vell. (Vellozo 1825 [1829]) are illegitimate homonyms of *Bignonia longisiliqua* Jacq. (Jacquin 1780). While *B. longisiliqua* Bertero ex Spreng. is a synonym of *M. obovata*, *Bignonia longisiliqua* Vell. is a synonym of *Catalpa longissima* (Jacq.) Dum. Cours., and *Bignonia longisiliqua* Jacq. is a synonym of *Stizophyllum perforatum* (Cham.) Miers. *Bignonia longisiliqua* Bertero ex Spreng. was described based on a specimen from “Ad fl. Magdalen.” (Magdalena river, Colombia) collected by C.L.G. Bertero. However, the referred specimen was never mentioned in subsequent taxonomic studies and could not be located. Instead, a specimen collected by C.L.G. Bertero in Santa Martha (Colombia) was cited by De Candolle (1845). *Doxantha longisiliqua* Miers is a new name subsequently published based on a specimen from “Sa. Martha” (Colombia), also collected by C.L.G. Bertero, with explicit reference to the specimen cited in De Candolle (1845). We found two specimens collected by Bertero in Santa Martha, one deposited at G-DC and the other deposited at MO. These specimens were identified as types of *Bignonia longisiliqua* Bertero ex Spreng., with the specimen at MO (barcode MO-074584) including a “Type Specimen” stamp. However, we did not find any publication citing the MO specimen as a nomenclatural type. In addition, it is likely that the specimen from Magdalena river that Sprengel used to describe *B. longisiliqua* was destroyed at B, where many specimens were held after 1890, including some of those collected by Bertero (Stafleu and Cowan 1976). Therefore, we designate the well-preserved specimen deposited at G-DC as a neotype for *Bignonia longisiliqua* Bertero ex Spreng. Lastly, the collection year written on the label “1822” is likely wrong because C.L.G. Bertero’s travels in the West Indies were from 1816–1821 (Stafleu and Cowan 1976). Given this information, we could not confidently assign the MO specimen as a duplicate (isoneotype).

In the original description of *Martinella gollmeri*, a specimen collected in Venezuela by J. Gollmer is cited. This specimen was most likely destroyed at B; no isotypes or illustrations have been found, and a neotype from Venezuela that matches the original description is here designated as a neotype.

The holotype of *Arrabidaea duckei* A.Samp. is interpreted to be mounted in two sheets, as both sheets have the same accession number (24094) written on their labels. Sheet 1 (RB-00536852) is a better material with leaves and an immature fruit.

#### Specimens examined.

**Belize**. **Cayo**: Hummingbird highway, south of Belmopan, vicinity of mile 28, premontane wet forest, 70 m, 14 Jun 1973, *A.H. Gentry 8250* (MO); Vicinity of Grano de Oro lumber camp south of Millionario, disturbed forest and roadside, 500 m, 2 Jun 1973, *A.H. Gentry 7767* (MO). **Bolivia**. **Beni**: José Ballivián, Comunidad Galilea, Canchon Tierra Negra, 200 m, 14°30'S, 66°37'W, 21 Oct 1994, E. Rivero 183 (SPF); Isla de Espiritu, Prov. Ballivian, Espiritu en la zona de influencia del río Yacuma. Sabana húmeda, 200 m, 13 Apr 1981, *St.G. Beck 5372* (MO). **La Paz**: Franz Tamayo, Madidi, Chalalan, Sendero Silvador, bosque amazonico preandino estacional, 350 m, 14°25'23"S, 67°55'26"W, 24 Nov 2004, A. Araujo-M 1532 (SPF); Prov. Nor Yungas, 4.1 km N of (below) Yolosa on road to Caranavi, secondary growth, roadside, 1200 m, 16°11'S, 67°44'W, 6 Oct 1984, *J.C. Solomon 12474* (MO). **Santa Cruz**: Prov. Ichilo, Parque Amboró, Río Semayo, Nueva Palestina, a 35 km al E de la Ciudad de Santa Cruz, 480 m, 17°45.5'S, 63°32'W, 4 Mar 1990, *R.C. Quevedo S. 51* (MO); Sara, Camino de Santa Rosa del Sara a Buen Retiro, 274 m, 17°13'57"S, 63°39'1"W, 7 Jan 2009, *G.A. Parada 1394* (SPF). **Brazil**. **Acre**: Bujari, margem direita do rio Antimary, sentido jusante a partir da ponte da BR-364, 154 m, 9°28'52.3"S, 68°20'55"W, 11 Dec 2016, *E.Y. Kataoka 406* (SPF); Mâncio Lima, São Domingos, Campina, solo arenoso humoso, 7°23'57"S, 72°45'41"W, 23 Oct 1998, *C.A.C. Ferreira 11751* (INPA); Rio Branco, próximo ao prédio do Herbário UFACPZ, ca. 60 m após fim da trilha de acesso do campus ao herbário, 182 m, 9°57'26.5"S, 67°52'27.8"W, 06 Dec 2016, *E.Y. Kataoka 390* (SPF). **Amapá**: Coastal Region survey, right bank of rio Flechal, 1°45'N, 50°58'W, 13 Aug 1962, *J.M. Pires 52504* (IAN, MG); Rio Araguarí, terra firme, baixa, 22 Jul 1951, *R.L. Fróes 27594* (IAN, R); Rio Araguari, along river between Mongubas and Serra do Navio, 0°42'N, 51°45'W, 25 Sept 1961, *J.M. Pires 51176* (IAN, INPA, MG); Rio Araguari, porto Platon, 18 Sept 1961, *J.M. Pires 51057* (IAN, MG); Matapi, mata baixa, terra firme, solo argiloso e úmido, 28 Dec 1976, *B.G.S. Ribeiro 1645* (MG); Porto Grande, Fazenda Governador, Aporema, 10 Nov 1982, *M. Dantas 1428* (IAN); Oiapoque: Amapá, Parque Nacional do Cabo Orange, igarapé Cova da Onça, 2°56'26.24"N, 50°59'3.147"W, 3 Aug 2006, S.R.M. Silva 62 (MG). **Amazonas**: Estrada Manaus-Caracaraí km 39, Reserva Experimental de Silvicultura Tropical, terra firme, solo arenoso, campina, 12 Sept 1977, *J. Ribamar 189* (INPA); Km 65, on road from Manaus to Boa Vista, manioc plantation, sandy soil, 22 Jul 1974, *A. Lasseign P21164* (INPA); Mindu, em capoeira, 5 Sept 1947, *T. Guedes 14* (IAN, RB); Rio Negro, próximo ao rio Arara, 2° acampamento da SIDERAMA, mata de terra firme, solo arenoso, 2 May 1973, *A.A. Loureiro 37946* (INPA, RB, SPF); Barcelos, Parque Nacional do Jaú, margem direita do Rio Negro, 35 m, 1°49'59.8"S, 61°29'38.5"W, 6 Jun 2016, *E.Y. Kataoka 247* (SPF); Rio Negro, between Ilha da Silva & Tapuruquara, 15 Oct 1971, *G.T. Prance 15281* (INPA); Humaitá, Estrada Humaitá-Lábrea, km 77, igarapé na beira da estrada, latossolo, 11 Jun 1982, *L.O.A. Teixeira 1076* (INPA); Itapiranga, Rio Uatumã, em frente ao igarapé Sta. Lizia, 1 km da margem direita do rio, mata de terra firme, solo argiloso, 16 Aug 1979, *C.A. Cid 448* (INPA); Lábrea, Rio Ituxi, vicinity of Boca do Curuquete, 8 Jul 1971, *G.T. Prance 14014* (INPA); Manaus, banks of rio Tarumã and Praia Dourada, sandy substrate, 14 Dec 1974, *A.H. Gentry 13292* (INPA); BR 17, igarapé da Bolívia, terra firme, arenoso, capoeira, 3 Jun 1955, *J. Chagas s.n.* (INPA, MG); Cabeceira do igarapé da Cachoeira Alta, terreno arenoso, capoeira, 28 Nov 1960, *W. Rodrigues 1956* (IAN, INPA); Campos Sales, igarapé da Cachoeira Alta do Tarumã, terra úmida, 22 Sept 1954, *J.C. de Almeida s.n.* (INPA); Campos Sales, km 10 da BR-17, terreno alagadiço, 30 Aug 1954, *J. Chagas s.n.* (INPA); Estrada da Forquilha solo arenoso, capoeira fechada, terra úmida, 11 Oct 1956, *J. Chagas s.n.* (INPA); Estrada do Aleixo, km 7 entrada à esquerda, terra firme, arenoso, capoeira aberta, 24 Aug 1956, *F. Mello s.n.* (INPA); Igarapé da Cachoeira Alta do Tarumã, terra firme, solo arenoso, capoeira, 17 Sept 1962, *W. Rodrigues 4635* (INPA); Igarapé da Cachoeira Alta, solo arenoso, úmido, capoeira, 11 Dec 1961, *W. Rodrigues 3846* (INPA); Km 10 da estrada do Aleixo, terra firme, arenoso, capoeira aberta, 14 Sept 1955, *D. Coelho s.n.* (INPA); Margem do igarapé da Bolívia, estrada da BR – 17, terra firme, arenosa, capoeira, 3 Jun 1955, *J.C. de Almeida s.n.* (INPA); Margem do igarapé da Bolívia terra firme, arenoso, capoeira baixa, aberta, 27 Jul 1956, *D. Coelho s.n.* (INPA); Margem do igarapé do Binda, terreno firme, arenoso, capoeira baixa, aberta, 27 Jul 1956, *D.F. Coelho 4001* (IAN); Margem do igarapé do Mariano terra firme, arenoso, capoeira, alta, 31 Jul 1956, *F.C. Mello 4016* (IAN, INPA); Margem do igarapé do parque 10, terra firme, arenoso, capoeira grossa, 6 Sept 1955, *F.C. Mello 1830* (INPA, MG); Parque 10 de Novembro, terra firme, úmido, arenoso, capoeira fechada, alta, 29 Feb 1956, *J. Chagas s.n.* (INPA); Ponta Negra, 11 Feb 1977, *M. Silva 2089* (INPA); Ponta Negra, campina de solo arenoso, 22 Jun 1961, *W. Rodrigues 2059* (INPA); Praia de Lajes, opposite meeting of the waters, Manaus, 11 Feb 1977, *G.T. Prance 24374* (INPA); Ramal Pau Rosa, na margem de um pequeno igarapé, 52 m, 02°50'42"S, 60°14'12"W, 11 Jun 2011, *R. Goldenberg 1554* (INPA); Reserva Florestal Adolpho Ducke, rodovia Manaus-Itacoatiara, km 26, lateral Oeste-Acará, floresta de baixio, 2°53'S, 59°58'W, 9 Aug 1995, *C.A. Sothers 558* (INPA, SPF); Reserva Florestal Adolpho Ducke, rodovia Manaus-Itacoatiara, km 26, saída do Igarapé Acará, área alterada, solo argiloso, próximo ao baixio/igarapé, 02°53'S, 59°58'W, 6 Jun 1997, *C.A. Sothers 1013* (INPA); Trilha próxima à entrada da Reserva Ducke pela AM-010 (km 26), ca. 300 m a partir da portaria da Reserva, trilha paralela à rodovia, 80 m, 2°54'49.72"S, 59°58'50.24"W, 22 Sept 2016, *E.Y. Kataoka 346* (SPF); Maués, rio Maués-Assu, lado oposto à cidade de Maués, capoeira de terra firme, solo argilo-arenoso, 3°23'S, 57°45'W, 21 Jul 1983, *C.A. Cid 4244* (INPA); Presidente Figueiredo, Balbina 193 km de Manaus, 12 Aug 1986, *C.A.A. Freitas 156* (INPA); Entorno, entrada à direita na estrada indo para a vila, depois da entrada para o CPPMA, 1°00'S, 59°00'W, 29 Nov 2006, *J.G. de Carvalho-Sobrinho 1246* (INPA, SPF); Entorno, picada da Suçuarana, 11 Jul 2007, *S. Sakagawa 435* (INPA, SPF); Rio Preto da Eva, 2–5 km N of Manaus-Itacoatiara road at Km 79 near Rio Preto da Eva, 100–200 m, 24 Nov 1974, *A.H. Gentry 12836* (INPA); São Gabriel da Cachoeira, road Camanaús-Uaupés near Camanaús, caatinga on white sand, terra firme, 1 Nov 1971, *G.T. Prance 15986* (INPA); Tabatinga, próximo ao aeroporto, sub-base do projeto RADAM/BRASIL, quadrícula SB-19xA, ponto 02, 1 May 1976, *C.D.A. Mota 358* (INPA, MG). **Goiás**: Alto Horizonte, Estrada para o trevinho, na ponte do rio Formiga, mata de galeria, 324 m, 14°9'1"S, 49°17'9"W, 1 May 2012, *J.E.Q. Faria* 2653 (SPF). **Maranhão**: Viana, Jan 1960, *O. de Carvalho 13* (RB); **Mato Grosso**: Aripuanã, Margem direita do rio Aripuanã, acesso pelo ‘Balneário Oásis’, acima da cachoeira, 224 m, 10°9'53.1"S, 59°27'44.9"W, 14 Nov 2016, *E.Y. Kataoka 380* (SPF); Cotriguaçu, Margem esquerda do rio Juruena, sentido montante, a partir da área de embarque da balsa, 192 m, 9°55'13.6"S, 58°15'4.2"W, 15 Nov 2016, *E.Y. Kataoka 381* (SPF); Rio Juruena, ilha próximo à margem esquerda do rio, 199 m, 9°54'2.8"S, 58°13'38.8"W, 15 Nov 2016, *E.Y. Kataoka 383* (SPF); Juara, margem direita do rio Apiacás, junto à primeira cachoeira, salto Apiacás, solo arenoso úmido, floresta aluvial, 26 May 1988, *M. Macedo 1918* (INPA). **Pará**: Aramanahy, low and high land, 11 Jan 1932, M.D. Cost 255 (IAN); Approx. 18 km east of Tucurui and rio Tocantins, by BR 263, white-sand campina and campina forest (campinarana), 3°30'S, 49°32'W, 28 Oct 1981, *D.C. Daly 1016* (INPA, MG); Beira do rio Mapuá, várzea, entre Vila Emilia e Boca do Mapuá, 18 Jul 1950, *G.A. Black 50-9804* (IAN); Beira do rio Mojú, fábrica e cereanías, 1 Jun 1954, *G.A. Black 54-16288* (IAN); Cacaual Grande, lado W do Canal Novais Filho, capoeira de várzea, 4 Jul 1952, *G.A. Black 52-15399* (IAN, RB); Km 15, Belem Brasilia highway E of Belem, near check point, 9 Dec 1974, *A.H. Gentry 13159* (IAN, INPA, MG); Km 56 da BR-163, *O.H. Knowles 1476* (MG); Região do Anapú, rio Pracajaí, Portel, 16 Sept 1956, *R.L. Fróes 32723* (IAN); Afuá, Rio Cajuuna, mata de várzea, margem inundável, 12 a 02 Sept – Oct 1992, *U.N. Maciel 1899* (MG); Alemquer, várzea, Amazonian Costa Rica, 28 Jul 1903, *A. Ducke s.n.* (MG); Anajás, Jun 1900, *A. Ducke s.n.* (MG); Aramanai, Belterra, Jan 1932, *R.C. Monteiro da Costa 255* (R); Aveiro, orla da mata firme, rio Tapajós, 2 Apr 1924, *J.G. Kuhlmann 1887* (R, RB); Barcarena, Itupanema, quintal, 23 Oct 1985, *M.C. Amorozo 220* (MG); Belém, Baixa da Pedreira, 3 Nov 1960, *E. Oliveira 1146* (IAN); Capoeira do Black, 25 m, 1°26'11.1"S, 48°26'37"W, 04 Nov 2016, *E.Y. Kataoka 360* (SPF); Capoeira do Black, 25 m, 1°26'11.1"S, 48°26'37"W, 04 Nov 2016, *E.Y. Kataoka 361* (SPF); Embrapa Amazônia Oriental, capoeira do Black, *A.C. da S. Andrade 141* (IAN); IPEAN grounds, forest edge and roadside second growth, alt. near sea level, 7 Dec 1974, *A.H. Gentry 13123* (IAN, INPA, MG); On lands of Instituto Agronômico do Norte, 2 K SO of Administration Building, near Rio Guamá, 15 Feb 1944, *A. Silva 117* (IAN); Parque do Utinga, aproximadamente 1,5 km da entrada do parque à direita, três metros da beira da estrada, 8 Jul 2011, *F.F.P. Castro s.n.* (MG); Benevides, rua na beira da BR316, no balneário Olho d’água, 1°21'19"S, 48°15'47"W, 31 Oct 2012, *M.P. do Nascimento 521* (IAN); Breves, local onde foi feito um inventário florestal, Oct-Nov 1957, *J.M. Pires 6653* (IAN); Gurupa, rio Moju, afluente do rio Amazonas, próximo ao porto de Gurupa, várzea alta, 8 Dec 1991, *G. dos Santos 314* (MG); Rio Moju, afluente do rio Amazonas, próximo ao porto de Gurupa, várzea alta, 8 Dec 1991, *G. dos Santos 317* (MG); Monte Alegre, Rio Maicurú, várzea, 12 Sept 1953, *R.L. Fróes 30203* (IAN); Oriximiná, rio Paru do oeste, mata de beira de rio, solo argiloso, 5 Sept 1980, *C.A. Cid 2155* (INPA, MG, RB); Ourém, 1°32'51"S, 47°6'36"W, Oct 2011, *F.C.A. Lucas 228* (IAN); Capoeira, 5 Dec 1903, *R.S. Rodr. s.n.* (MG); Paragominas, Rodovia Belém-Brasília. Rio Uraim, beira do rio, terreno alagado, 18 Jan 1966, *M. Silva 452* (MG); Porto Trombetas, mineração rio do norte, 1991, *E. Soares 545* (INPA); Mineração rio do norte, 1991, *E. Soares 766* (INPA); Mineração rio do norte, restinga do rio Trombetas 1991, *E. Soares 345* (INPA); Mineração rio do norte, restinga do rio Trombetas, 3 Jul 1991, *O.H. Knowles 1732* (INPA); Primavera, Subindo o rio Quatipuru, aproximadamente 5 km da ilha de Maçaranduba, capoeira baixa, 24 Nov 1993, *R. Lisboa 2992* (MG); Santarém, Km 35 da estrada do Palhão, arredores do acampamento do igarapé Curupira, capoeira, beira do igarapé, 30 Aug 1969, *M. Silva 2446* (MG); Rio Curuaúna, várzea inundável, região do planalto de Santarém onde foi feito o levantamento estatístico florestal pelo IAN, SPVEA e FAO, Oct 1954, *R.L. Fróes 31360* (IAN); Tapeirinha para Santarém, terra firme, 25 Dec 1958, *F. Markgraf 3872* (RB); Vitória do Xingu, Usina Hidrelétrica de Belo Monte, 19 Jun 2012, *L.S. Lima 417* (MG). **Rondônia**: Porto Velho, BR 364, Rio Novo próximo ao acesso à UHE de Samuel, mata secundária, solo argilo-arenoso, 16 Aug 1987, *A. Vasques 18* (INPA); Mata ciliar, UHE – Samuel, Rio Jamari, montante da UHE, margem direita, 10 May 1988, *M. Pereira 230* (RB). **Roraima**: Boa Vista, T.F. de Roraima, terra firme, beira do rio, 20 Feb 1964, *M. Silva 16* (IAN, MG); Caracaraí, margem direita do Rio Branco, 26 m, 1°2'50.3"S, 61°52'6.7"W, 8 Jun 2016, *E.Y. Kataoka 273b* (SPF); Rorainópolis, confluência Rio Negro/Rio Branco, margem esquerda do Rio Branco, 33 m, 1°23'30.2"S, 61°50'20.7"W, 7 Jun 2016, *E.Y. Kataoka 250* (SPF); Foz do rio Branco no rio Negro, mata de igapó com interferência da água branca do rio Branco, 1°23'27.1"S, 61°50'31.6"W, 13 May 2015, *B.M. Gomes 647* (SPF); Margem a jusante do rio Branco, borda de mata de várzea, 22 m, 1°4'41.92"S, 61°51'7.33"W, 8 Jun 2016, *A. Frazão 267* (SPF); Margem esquerda do Rio Branco, 27 m, 1°17'42.2"S, 61°51'1.7"W, 10 Jun 2016, *E.Y. Kataoka 330* (SPF); Margem esquerda do Rio Branco, 28 m, 0°38'47.7"S, 61°49'15.3"W, 9 Jun 2016, *E.Y. Kataoka 309* (SPF); Margem esquerda do Rio Branco, 28 m, 0°38'47.9"S, 61°49'15.5"W, 9 Jun 2016, *E.Y. Kataoka 310* (SPF); Margem esquerda do Rio Branco, 28 m, 0°38'54.1"S, 61°49'18.2"W, 9 Jun 2016, *E.Y. Kataoka 311* (SPF); Margem esquerda do Rio Branco, 28 m, 0°38'54.1"S, 61°49'18.2"W, 9 Jun 2016, *E.Y. Kataoka 312* (SPF); Margem esquerda do Rio Branco, 28 m, 0°50'30.3"S, 61°51'4.6"W, 8 Jun 2016, *E.Y. Kataoka 289* (SPF); Margem esquerda do Rio Branco, 28 m, 0°50'30.3"S, 61°51'4.6"W, 8 Jun 2016, *E.Y. Kataoka 290* (SPF); Margem esquerda do Rio Branco, 28 m, 1°16'41.8"S, 61°50'13.9"W, 10 Jun 2016, *E.Y. Kataoka 329* (SPF); Margem esquerda do Rio Branco, 28 m, 1°5'46.9"S, 61°52'7.4"W, 10 Jun 2016, *E.Y. Kataoka 324* (SPF); Margem esquerda do Rio Branco, 29 m, 1°23'9.4"S, 61°50'54.4"W, 7 Jun 2016, *E.Y. Kataoka 251* (SPF); Margem esquerda do Rio Branco, 30 m, 0°39'20.1"S, 61°49'32.1"W, 9 Jun 2016, *E.Y. Kataoka 313* (SPF); Margem esquerda do Rio Branco, 31 m, 1°23'2.8"S, 61°51'3.7"W, 7 Jun 2016, *E.Y. Kataoka 252* (SPF); Margem esquerda do Rio Branco, 31 m, 1°23'22.9"S, 61°50'38"W, 10 Jun 2016, *E.Y. Kataoka 332* (SPF); Margem esquerda do Rio Branco, 33 m, 0°40'57.1"S, 61°50'37.8"W, 8 Jun 2016, *E.Y. Kataoka 302* (SPF); Margem esquerda do Rio Branco, 34 m, 1°20'39.7"S, 61°52'5.2"W, 7 Jun 2016, *E.Y. Kataoka 262* (SPF); Margem esquerda do Rio Branco, 37 m, 0°50'32.4"S, 61°51'5.7"W, 8 Jun 2016, *E.Y. Kataoka 288* (SPF). **Colombia**. **Amazonas**: Leticia, Parque Nacional Natural Amacayacu, cerca de la Cabaña en la boca del río Amacayacu, bosque secundario, 14 Jun 1992, *R. Rueda 525* (MO). **Antioquia**: Cáceres, 10–15 km NE de Cáceres en la Troncal de la paz, 180 m, 7°40'N, 75°22'W, 6 Nov 1987, *R. Callejas 5380* (MO). **Bolivar**: Isla de Barú, entre Santa Ana y Playa Mojana, 20 m, 25 Aug 1986, *H. Cuadros 3070* (MO); Road to Pta. Barú, W of Cartagena across Canal de Dique, scrubby (dry) forest remnants, 20 m, 10°18'N, 75°35'W, 4 Jul 1984, *A.H. Gentry 47648* (MO); Cartagena, Along road ca. 7 km SW of Arroyo Grande, old secondary vegetation, 70 m, 10°36'N, 75°24'W, 31 Jul 1985, *J.L. Zarucchi 3901* (MO); Morales, Cgto Norosí, caminoa Tiquisionuevo, 200 m, Apr 1985, *H. Cuadros 2126* (MO); San Juan, Loma de los colorados, 2 km S of San Juan, disturbed moist forest, 300 m, 9°58'N, 75°10'W, 27 Oct 1989, *A.H. Gentry 68258* (MO); Turbaco, Camino al depositario, 100 m, 8 Jun 1982, *H.C. Villalobos 1373* (MO). **Córdoba**: Ayapel, Carreteable a Ayapel de la Carretera Caucasia – Nechi, Finca del diamante, 50 m, 8°12'N, 74°47'W, 5 May 1999, *F.J. Roldán 2807* (MO); Junction of río Tigre and río manso, Paramillo National Park, transect no. 6, 200 m, 7°30'N, 76°5'W, 28 Jul 1988, *A.H. Gentry 63841* (MO). **Caquetá**: Solano, Sitio Araracuara, pista de aterrizaje, colecciones sobre afloramiento rocoso, 200 m, 0°35'S, 72°25'W, 10–25 May 1998, *M.V. Arbeláez 1088* (MO). **Chocó**: Riosucio, Peye, orillas del río Peye, 30 m, 5 Jun 1976, *E. Forero 1874* (INPA). **Sucre**: San Onofre, corregimiento “Las Brisas”, clto de “Salas”, Arroyo “Mambú”, 11 Sept 1996, *A. Realpe 229* (SPF). **Valle**: Bajo Calima, pluvial forest, road to Juanchaco Palmeras, 100 m, 3°55'N, 77°2'W, 10 Jul 1984, *A.H. Gentry 47843* (MO). **Costa Rica**. **Alajuela**: San Isidro de San Ramón, 1259 m, 10°4'46"N, 84°26'30"W, 25 Oct 1986, *G. Herrera 123* (MO); Cantón de Upala, Z.P. Tenorio, cordillera de Guanacaste, Bijagua, primary forest on a ridge at Volcán Tenorio, premontane rainforest, 1000–1500 m, 10°43'0"N, 85°1'0"W, 20 Apr 1995, *D. Penneys 485* (MO). **Guanacaste**: Liberia, P.N. Guanacaste, cuenca del Tempisque, estación Cacao, alrededores de la estación, 1100 m, 10°55'36"N, 85°28'6"W, 7 Aug 2007, *A. Soto 1822* (MO); P.N. Guanacaste, cordillera de Guanacaste, Estación Cacao, Sendero Maritza, 1100 m, 10°55'43"N, 85°28'10"W, 9 Feb 1995, *L. Angulo 42* (MO); P.N. Guanacaste, cuenca del Tempisque sector Cacao, hacia la estacíon de 1.5 km, después del río Góngora, 650 m, 10°53'10.421"N, 85°28'19"W, 30 Apr 2000, *L. Acosta 1057* (MO); P.N. Rincón de la Vieja, cuenca del Tempisque, sendero hacia el cráter, 1004 m, 10°47'19.3"N, 85°20'57"W, 31 May 2011, *L.D. Vargas 4556* (MO); Parque Nacional de Guanacaste estación Cacao, 1100 m, 10°55'45"N, 85°28'15"W, 31 Oct 1993, *C. Chávez 332* (MO); Parque Rincón de La Vieja, del mirador siguiendo la fila al volcán Santa María, 1100 m, 10°46'N, 85°49'W, 22 Nov 1987, *G. Herrera 1364* (MO). **Heredia**: La Selva, Río Sarapiqui near Puerto Viejo, tropical wet forest, junction SSO and LOC trails, 100 m, 10°26'N, 84°1'W, 8 Jan 1993, *A.H. Gentry 78642* (MO). **Puntarenas**: Monteverde, Pacific slope forest, 1450 m, 8 Aug 1985, *W.A. Haber 2204* (MO); Monteverde, Pacific slope forest, 1450 m, 8 Aug 1985, *W.A. Haber 2211* (MO); Golfito, P.N. Corcovado, peninsula de Osa, Estacion Sirena, sendero Espaveles, 0 m, 8°28'51"N, 83°35'42"W, 16 Jan 1997, *R. Aguilar 4986* (MO); Refugio de Vida Silvestre Golfito, camino a la Gamba, 94 m, 8°40'56"N, 83°11'50"W, 9 Oct 2008, R. Aguilar 11418 (MO, SPF); San Luis, Monteverde, camino a Veracruz, 1050 m, 10°16'35"N, 84°47'45"W, 16 Oct 1992, *A. Fernández 440* (MO). **San Jose**: Reserva Biológica Carara, sector Bijagual, sitio Sendero Bijagual-Quebrada Bonita, 550 m, 9°46'20"N, 84°33'50"W, 1 Nov 1990, *R. Zúñiga 327* (MO); Cantón de Dota, faja costeña del Valle de Parrita, faldas Cerro Narra, quebrada Salitrillo, bosque primario cerca del cauce, 200 m, 9°28'55"N, 84°2'50"W, 19 Jul 1995, *J.F. Morales 4572* (MO). **Ecuador. Cantón Archidona**: Napo, Faldas al sur del Volcán Sumaco, carretera Hollin-Loreto, km 31, comuna Challua Yacu, bosque pluvial pre montano, suelos de origen volcánico, 1200 m, 0°43'S, 77°36'W, 15–17 Nov 1988, *A. Alvarado 46* (MO). **Los Ríos**: Hacienda Los Ocho, km 50 on road from Santo Domingo to Quevado, wet Forest, 200 m, 4 Feb 1974, *A.H. Gentry 9640* (QCA). **Napo**: Archidona Cantón, faldas al sur del Volcán Sumaco, carretera Hollin-Loreto, km 31, comuna Challua Yacu, bosque pluvial pre montano, suelos de origen volcánico, 1200 m, 0°43'S, 77°36'W, 15–17 Nov 1988, *A. Alvarado 46* (QCNE). **Orellana**: Along MAXUS (YPF) road at Km 50, Yasuni National Park, 250 m, 16 Mar 1997, *R.J. Burnham 1493* (QCNE). **Pastaza**: Shell, Vicinity of Shell, 1.2 km N of town, disturbed virgin forest in swampy area, 1092 m, 1°29'33"S, 78°3'57"W, 9 May 2003, *T.B. Croat 88873* (QCNE). **Pichincha**: Quito Cantón, Parroquia Puerto Quito, reserva Florestal de ENDESA, 10 km al norte de Alvaro Pérez Intriago, bosque muy húmedo premontano, bosque primario, transectos, 650–800 m, 00°03'N, 79°07'W, 11 Jun 1990, *C.E. Cerón 10140* (MO, QCNE). **Sucumbíos**: Lago Agrio Cantón, Reserva Cuyabeno, laguna Grande, bosque húmedo tropical, bosque primario sobre suelo bien drenado alrededor de cabañas de Neotropic, 230 m, 0°0'S, 76°11'W, 15 Nov 1991, *W. Palacios 9153* (QCNE). **Zamora-chinchipe**: Nangaritza Cantón, Mizai, in río Nangaritza valley, forest on slope above military post, 850 m, 4°18'S, 78°40'W, 31 Jul 1993, *A.H. Gentry 80967* (QCNE). **French Guiana**. Montagne de Kaw, borde de piste forestière, 40 m, 4°33'N, 52°9'W, 25 Mar 1988, *G. Cremers 9827* (MO); Cayenne, Ile de Cayenne, bord de route, la rocade, entre les ronds points Baduel et la Madeleine à proximité du croisement avec la route Raban, 7 m, 4°56'N, 52°20'W, 9 Mar 2009, *C. Delnatte 1693* (MO). **Saül**: La Fumée Mountain, Antenne Nord, non-flooded moist forest, 400 m, 3°37'N, 53°12'W, 14 May 1986, *S.A. Mori 17998* (MO). **Guyana**. Bords de la Rivière du Maroni 1861, *M. Melinon 13* (R); Kariako village, Barama river, North-West District Mora riparian forest around Kariako village, 145 m, 7°22'N, 59°42'W, 23 Dec 1995, *T. van Andel 680* (MO). **Honduras**. **Atlántida**: Campamento Quebrada Grande ca. 10 km south west of La Ceiba, at base of north slope of Pico Bonito, from camp to 2 km east of camp, upland forest on slope, 80 – 180 m, 15°42'N, 86°51'W, 10 May 1993, *R.L. Liesner 26180* (MO). **Yoro**: Cordillera Nombre de Dios, hills S of San José de Texíguat, evergreen rainforest on steep slopes, 350 m, 15°29'N, 87°26'W, 17 May 1991, *G. Davidse 34509* (MO). **Mexico. Veracruz**: Hidalgotitlán, afluente O del río Las Cuevas, +/- 7–9 horas a pie al S del de La Laguna, área arriba de las cascadas donde el arroyo corre en direccíon E-O, entre lomas con suelos prof., selva alta perenifolia con mucha jimba, 350 m, 17°13'30"N, 94°30'30"W, 17 Apr 1982, *T. Wendt 3865* (MO). **Peru. Amazonas**: Bagua, Distrito Imaza, Comunidad de Yamakat, bosque primario, 600 m, 5°3'24"S, 78°20'17"W, 6 Jun 1997, *R. Vásquez 23907* (MO). **San Martín**: Prov. Mariscal Caceres, Dtto Tocache Nuevo, al sud oeste del Aeropuerto de Tocache Nuevo, en bosque secundario, 400 m, 12 Jan 1970, *J. Schunke V. 3691* (INPA). **Loreto**: Km 22 Yurimaguas-Tarapoto road, remnant patch of mature forest on white sand and adjacent scrub, 190 m, 6°S, 76°13'W, 10 Oct 1985, *A.H. Gentry 52177* (MO); Iquitos, Laguna Quistococha, ca. 15 km SW of Iquitos, 8 July 1977, *J.C. Solomon 3448* (MO). **Madre de Dios**: Tobapata Province, Cuzco Amazónico Lodge, lago Sandobal and río Madre de Dios, lake edge, aguajal, 200 m, 12°35'S, 69°3'W, 14 Apr 1990, *P. Núñez 12071* (MO). **Maynas**: Alpahuayo (km 25, carretera Iquitos-Nat.), Estacíon IIAP, bosque primario, 19 Oct 1984, *R. Vásquez 5769* (MO); Iquitos, Río Itaya, Sanangal, restinga over silty clay mostly disturbed, 110 m, 17 May 1980, *S. McDaniel 23739* (MO); Río Nanay, Carretera de Picuruyacu, en terreno arenoso, 160 m, 23 Sept 1981, *M. Rimachi Y. 5720* (MO); Nauta, Carretera a Iquitos, bosque inundable estacional, 150 m, 4°29'S, 75°35'W, 12 Dec 1986, *R. Vásquez 8604* (MO); Pebas, Río Ampiyacu, 19 Jul 1976, *J. Revilla 924* (MO). **Pasco**: Oxapampa, Dist. Palcazú, comunidad nativa Alto Lagarto – Reserva Comunal Yanesha, remanente de bosque primario, 584 m, 10°9'7"S, 75°23'32"W, 30 Oct 2009, *R. Rojas 7122* (SPF); Distrito Palcazú. Comunidad Nativa Centro Connás, bosque primario remanente en borde de carretera, 373 m, 10°9'59"S, 75°16'5"W, 14 May 2010, *R. Vásquez 36497* (SPF). **Tocache Nuevo**: San Martín, al sud oeste del Aeropuerto de Tocache Nuevo, en bosque secundario, 400 m, 12 Jan 1970, *J. Schunke V. 3691* (IAN). **Suriname. Brokopondo**: NW of Brokopondo Stuwmeer Lake (E of Brownsberg Nature Reserve), Tonka island, trail west from main compound, high forest on laterite soil, 15 m, 4°35'N, 55°7'W, 4 Feb 1999, *B. Hoffman 5299* (MO); Brownsweg, near Brownsweg Nature Park, scarcely disturbed high mesophytic rain forest on slope, gravelly clay soil, 240 m, 4°35'41.28"N, 55°6'0"W, 7 Oct 2005, *S. Ruysschaert SRU 728* (SPF). **Paramaribo**: Bakboord farm and lake property, just within NE corner of Paramaribo city limits, ca. 1 km N of Kwattaweg, ca. 1/2 km E of Henri Fernandesweg, secondary forest patch bordering fairly recently abandoned pastureland, 5°50'60"N, 55°12'59.97"W, 25 Nov 1996, *R.J. Evans 2591* (MO, RB). **Sipaliwini**: Kwamalasemutu village vicinity, 50 m, 2°22.5'N, 56°47.3'W, 23 Feb 1999, *M.J. Plotkin 1359* (MO). **Venezuela**. Delta Amacuro, cienega de selva húmeda caliente y selva de galería, carretera Caño Guará – La Horqueta, 30–31 Jan 1982, *B. Stergios 3982* (MO). **Amazonas**: Road from San Fernando do Atapabo to Santa Barbara 12–40 km from San Fernando, thickets and forest, mostly on white sand, 110 m, 24 Mar 1974, *A.H. Gentry 10964* (MO); Atures, bosque húmedo del río Cataniapo entre San Pedro de Cataniapo y comunidad El Milagro, 95 m, 6°25'N, 67°25'W, 12 Aug 1986, *A. Castillo 2179* (MO); Primary rainforest, along road between Paso el Diablo and Caño de Culebra, 25–30 km southeast of Puerto Ayacucho, 100 m, 12 May 1980, *J.A. Steyermark 122336* (MO). **Bolivar**: Reserva Florestal Itamaca, selva pluvial del bajo río Botanamo, entre su desemboque al río Cuyuni hasta la boca del río Guarapín, 16–17 Jul 1983, *B. Stergios 6108* (MO).

### 5. Martinella tomentosa

Taxon classificationPlantaeLamialesBignoniaceae

Kataoka & L.G.Lohmann sp. nov.

5E486283-1E67-5403-AD05-AECDCC681B4F

urn:lsid:ipni.org:names:77217117-1

Fig. 11

#### Type.

Brazil. Amazonas: Manaus, Reserva Florestal Adolfo Ducke, Rodovia Manaus Itacoatiara, km 26, 02°53'S, 59°58'W, 19 Jun 1995, *M.A.D de Souza & C.F. da Silva*, *39* (holotype: SPF-102015!; isotypes: INPA-179938!, MO not seen).

#### Diagnosis.

*Martinella tomentosa* differs from other Amazonian species of *Martinella* by the tomentose leaflets and branches, and inflorescences arranged in thyrses, as opposed to the glabrous to lanuginose leaflets and inflorescences arranged in racemes or lax thyrses of all other Amazonian species.

**Figure 11.**
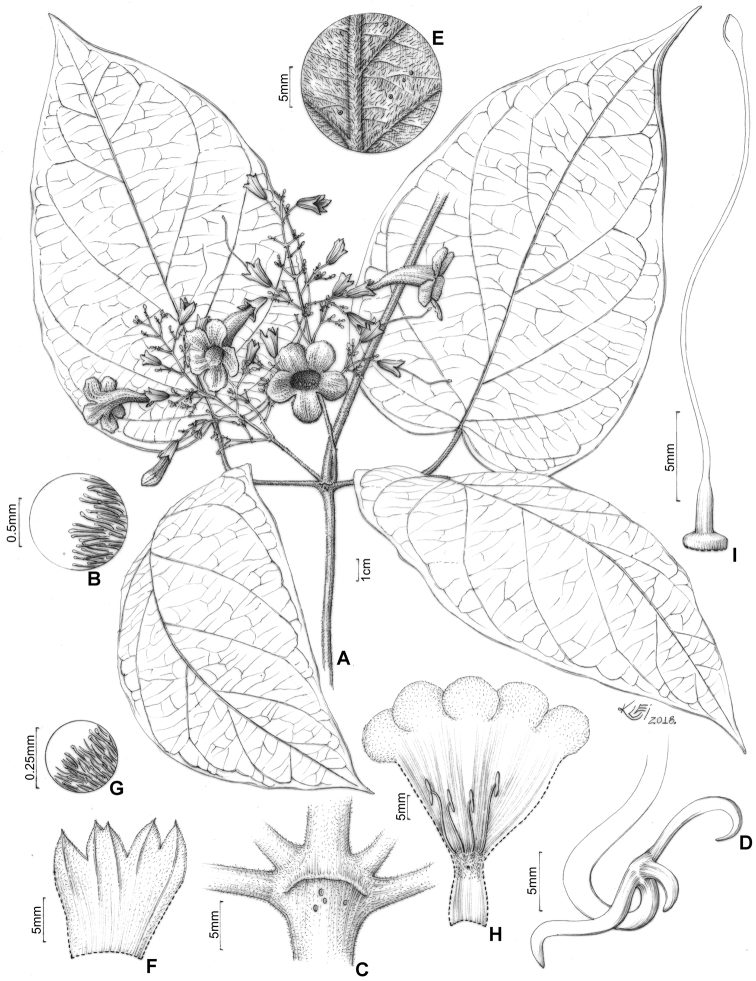
*Martinella tomentosa* Kataoka & L.G. Lohmann **A** flowering branch **B** leaflet indumentum **C** ridge at the interpetiolar region and patelliform glandular trichomes **D** trifid tendril **E** abaxial side of leaflet with patelliform glandular trichomes **F** calyx external view **G** calyx indumentum **H** open flower showing anthers, trichome distribution, and reduced (ca. 1 mm) staminode **I** gynoecium. Illustrated by Klei Sousa, based on *M.A.D. de Souza 39* (**A–C, F–I**), SPF; *W. Rodrigues 4444* (**E**), *M.F. Silva 855* (**D**), INPA.

#### Description.

**Lianas**; ***branches*** with solid pith, cylindrical, green, drying brown, smooth, tomentose, densely covered with simple eglandular trichomes and stipitate glandular trichomes, with scattered patelliform glandular trichomes more frequently at interpetiolar region; prophylls of the axillary buds densely covered with simple eglandular trichomes and stipitate glandular trichomes. ***Leaves*** 2-foliolate, with the terminal leaﬂet generally modified into a trifid tendril; petioles terete, not pulvinated, 38.1–58.6 mm long, densely covered with simple eglandular trichomes and stipitate glandular trichomes with few scattered patelliform glandular trichomes; petiolules terete, pulvinated, 27–31.5 mm long, densely covered with simple eglandular trichomes and stipitate glandular trichomes with occasional patelliform glandular trichomes; leaﬂets concolorous, chartaceous, ovate, apex acuminate, base cordate, margins entire and slightly revolute, 16.5–19.0 × 12.0–14.0 cm, adaxial surface glabrescent with simple eglandular trichomes and stipitate glandular trichomes at the canaliculi of veins, abaxial surface tomentose, densely covered with simple eglandular trichomes and stipitate glandular trichomes, and patelliform glandular trichomes concentrated near the base and few scattered along the midvein. ***Inﬂorescences*** in thyrses; 10–19.5 cm long, tomentose, densely covered with simple eglandular trichomes and stipitate and few patelliform glandular trichomes; bracts linear, ca. 2 mm long, pubescent, densely covered with simple eglandular trichomes and stipitate glandular trichomes; pedicels terete, 4.3–9.4 mm, pubescent, with simple eglandular trichomes and stipitate glandular trichomes. ***Flowers*** with calyx green, chartaceous, campanulate, 14–21.8 × 18.1–21.3 mm, densely covered with simple eglandular trichomes and stipitate glandular trichomes, with few patelliform glandular trichomes, lobes 2–4, apex acuminate, pubescent; corolla white to lilac, membranous, 44.9–48.7 mm long, narrowly tubular basal portion 14.5–14.9 mm long × 2.9–3.2 mm wide, upper campanulate portion 28.4–30.2 mm long × 12.1–14.9 mm wide, lobes subcircular, 6.5–7.8 × 11.8–12.1 mm; stamens in two lengths, longer ones 14.4–15.4 mm, shorter ones 10.3–10.5 mm, thecae 2.6–3.1 mm, glabrous; staminode not seen; gynoecium 28.4–36.3 mm long; ovary glabrous; style glabrous; stigma lanceolate, glabrous; nectariferous disk 2.3–2.5 × 1–1.1 mm. ***Fruits and seeds*** not seen.

#### Distribution and habitat.

**Figure 12.**
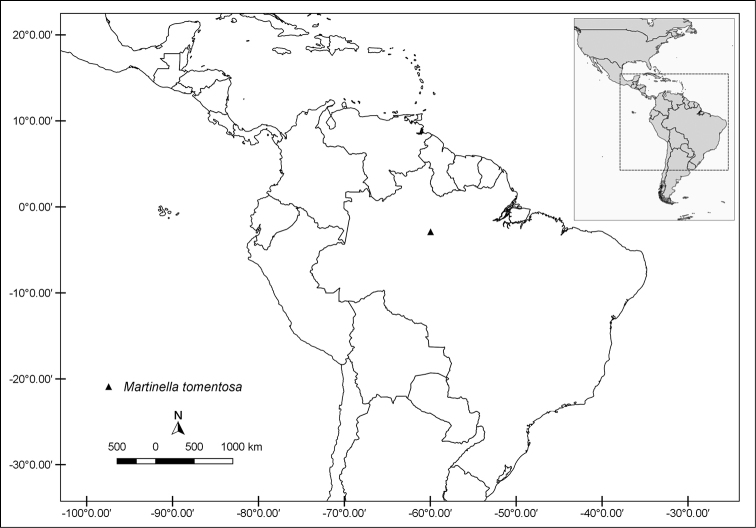
Distribution map of *Martinella tomentosa*.

*Martinella tomentosa* is restricted to the central portion of Amazonia (Fig. 12), where it occurs in *Terra Firme* forests from Brazil (state of Manaus).

#### Etymology.

The species epithet relates to the distinguished tomentose indumentum in leaves and branches of *M. tomentosa*.

#### Phenology.

Flowering specimens were collected in June.

#### Conservation status.

Data deficient (DD); known from only three specimens of two localities.

#### Discussion.

*Martinella tomentosa* is a new species whose description is strongly supported by morphological and molecular phylogenetic data (Kataoka and Lohmann in prep). The tomentose leaves and branches are the most striking characteristics that easily distinguish *M. tomentosa* from all other species of Amazonian *Martinella*. This new taxon is only known from very few collections from Central Amazonia, none of which was collected during the fruiting season.

#### Specimens examined.

**Brazil. Amazonas**: Estrada Castanho-Tupana, entre o km 50–40, solo argiloso, margem da estrada sempre alagada, 18 Jul 1972, *M.F. Silva 855* (INPA); Manaus, Igarapé do Passarinho, terreno firme, argiloso, capoeira grossa, 15 May 1962, *W. Rodrigues 4444* (INPA); Reserva Florestal Adolfo Ducke, Manaus Itacoatiara, km 26, 2°53'S, 59°58'W, 28 Sept 1995, *C.D. Leme 39* (INPA).

## Supplementary Material

XML Treatment for Martinella

XML Treatment for Martinella insculpta

XML Treatment for Martinella insignis

XML Treatment for Martinella lanuginosa

XML Treatment for Martinella obovata

XML Treatment for Martinella tomentosa

## Supplementary materials

Supplementary material 1 Index to numbered collections Data type Index to numbered collection. Explanation note The file contains a list of all numbered collections analysed for the taxonomic revision https://binary.pensoft.net/file/542009This dataset is made available under the Open Database License (http://opendatacommons.org/licenses/odbl/1.0/). The Open Database License (ODbL) is a license agreement intended to allow users to freely share, modify, and use this Dataset while maintaining this same freedom for others, provided that the original source and author(s) are credited.Eric Y. Kataoka, Lúcia G. Lohmann
