# Supplementary material for: Taxonomic revision of Martinella Baill. (Bignonieae, Bignoniaceae)
Source: PhytoKeys. 2021 May 13;177:77–116. doi: 10.3897/phytokeys.177.64465 (PMC8137638; doi:10.3897/phytokeys.177.64465)
Supplement: Supplementary material 1 — Index to numbered collections [file phytokeys-177-077-s001.docx]

# Appendix

**Index to numbered collections**

A. Alvarado 46 (obovata).

A. Araujo-M 1532 (obovata).

A. Castillo 2179 (obovata).

A. Ducke s.n. (obovata).

A. Estrada 3072 (insculpta).

A. Fernández 440 (obovata).

A. Frazão 267 (obovata).

A. Lasseign P21164 (obovata).

A. Loureiro 37901 (insculpta); s.n. (obovata).

A. Realpe 229 (obovata).

A. Rodríguez 3367 (insculpta).

A. Silva 117 (obovata).

A. Soto 1822 (obovata).

A. Vasques 18 (obovata).

A.A. Loureiro 37946 (obovata).

A.C. da S. Andrade 141 (obovata).

A.H. Gentry 11208 (insculpta); 28200 (insculpta); 41384 (insculpta); 27233 (lanuginosa); 52144 (lanuginosa); 7767 (obovata); 8250 (obovata); 9640 (obovata); 10964 (obovata); 12836 (obovata); 13024 (insculpta); 13123 (obovata); 13159 (obovata); 13292 (obovata); 47648 (obovata); 47843 (obovata); 52177 (obovata); 63841 (obovata); 68258 (obovata); 78642 (obovata); 80967 (obovata).

A.R. Zuntini 151 (insignis); 321 (insignis).

B. Boyle 7052 (insculpta).

B. Hoffman 5299 (obovata).

B. Stergios 3982 (obovata); 6108 (obovata).

B.G.S. Ribeiro 1645 (obovata).

B.L. Stannard 423 (insculpta).

B.M. Gomes 647 (obovata).

B.V. Rabelo 2386 (insculpta).

C. Chávez 332 (obovata).

C. Delnatte 1693 (obovata).

C.A. Cid 448 (obovata); 2155 (obovata); 4244 (obovata).

C.A. Sothers 558 (obovata); 1013 (obovata).

C.A.A. Freitas 156 (obovata).

C.A.C. Ferreira 11751 (obovata).

C.A.W. Schwacke 425 (insculpta); 463 (insculpta).

C.D. Leme 39 (tomentosa).

C.D.A. Mota 358 (obovata).

C.E. Cerón 10140 (obovata).

D. Coelho s.n. (obovata).

D. Penneys 485 (obovata).

D.C. Daly 1016 (obovata).

D.F. Coelho 4001 (obovata).

E. Forero 1874 (obovata).

E. Oliveira 1146 (obovata).

E. Rivero 183 (obovata).

E. Soares 345 (obovata); E. Soares 545 (obovata); E. Soares 766 (obovata).

E.Y. Kataoka 339 (insculpta); 342 (insculpta); 344 (insculpta); 370 (insculpta); 372 (insculpta); 404 (insculpta); 407 (insculpta); 247 (obovata); 250 (obovata); 251 (obovata); 252 (obovata); 262 (obovata); 273b (obovata); 288 (obovata); 289 (obovata); 290 (obovata); 302 (obovata); 309 (obovata); 310 (obovata); 311 (obovata); 312 (obovata); 313 (obovata); 324 (obovata); 329 (obovata); 330 (obovata); 332 (obovata); 346 (obovata); 360 (obovata); 361 (obovata); 380 (obovata); 381 (obovata); 383 (obovata); 390 (obovata); 406 (obovata).

F. Ayala 1793 (insculpta).

F. de C. Mello 1830 (obovata).

F. Guanchez 923 (insculpta).

F. Markgraf 3872 (obovata).

F. Mello s.n. (obovata).

F.C. Mello 4016 (obovata).

F.C.A. Lucas 228 (obovata).

F.F.P. Castro s.n. (obovata).

F.J. Roldán 2807 (obovata).

G. Cremers 9827 (obovata).

G. Davidse 34509 (obovata).

G. dos Santos 314 (obovata); 317 (obovata).

G. Herrera 123 (obovata); 1364 (obovata).

G. McPherson 20083 (insculpta).

G.A. Black 50-9804 (obovata); 52-15399 (obovata); 54-16288 (obovata).

G.A. Parada 1394 (obovata).

G.T. Prance 14717 (insculpta); 14914 (insculpta); 15557 (insculpta); 14014 (obovata); 15281 (obovata); 15986 (obovata); 24374 (obovata).

H. Cuadros 2126 (obovata); 3070 (obovata).

H.C. Villalobos 1373 (obovata).

I. Huamantupa 3698 (lanuginosa).

J. Chagas s.n. (obovata).

J. Revilla 924 (obovata); 979 (insculpta).

J. Ribamar 189 (obovata).

J. Schunke V. 3691 (obovata).

J.A. Steyermark 104035 (insculpta); 122336 (obovata).

J.A.C. da Silva 1294 (insculpta).

J.C. de Almeida s.n. (obovata).

J.C. Solomon 6108 (insculpta); 3448 (obovata); 12474 (obovata).

J.E.Q. Faria 2653 (obovata).

J.F. Morales 910 (insculpta); 4572 (obovata).

J.G. de Carvalho-Sobrinho 1246 (obovata).

J.G. Kuhlmann 1887 (obovata).

J.J. Hernandez 99 (insculpta).

J.L. Zarucchi 3901 (obovata).

J.M. Pires 6653 (obovata); 51057 (obovata); 51176 (obovata); 52504 (obovata).

K. Van Kerckhove MVK 114 (insculpta).

L. Acosta 1057 (obovata).

L. Angulo 42 (obovata).

L. Ferreira 109 (lanuginosa).

L.D. Vargas 4556 (obovata).

L.G. Lohmann 616 (lanuginosa).

L.O.A. Teixeira 958 (insculpta); 1076 (obovata).

L.S. Lima 417 (obovata).

M. Dantas 1428 (obovata).

M. Goulding 1324 (insculpta).

M. Macedo 1918 (obovata).

M. Melinon 13 (obovata).

M. Pereira 230 (obovata).

M. Rimachi Y. 835 (insculpta); 6180 (insculpta); 6270 (insculpta); 5720 (obovata).

M. Silva 16 (obovata); 452 (obovata); 2089 (obovata); 2446 (obovata).

M.A.D. de Souza 39 (tomentosa).

M.C. Amorozo 220 (obovata).

M.d. Cost 255 (obovata).

M.F. Silva 855 (tomentosa).

M.J. Plotkin 1359 (obovata).

M.P. do Nascimento 521 (obovata).

M.V. Arbeláez 1088 (obovata).

N.Y.Bot. Garden 51176 (obovata).

O. de Carvalho 13 (obovata).

O.H. Knowles 1476 (obovata); 1732 (obovata).

O.H. Knowles 1732 (obovata).

P. Núñez 12071 (obovata).

P.L. Lisboa 6 (insculpta).

R. Aguilar 4986 (obovata); 11418 (obovata).

R. Callejas 1629 (insignis); 5380 (obovata).

R. Goldenberg 1554 (obovata).

R. Hahn 123 (insculpta).

R. Liesner 15711 (insculpta); 16956 (insculpta).

R. Lisboa 2992 (obovata).

R. Rojas 7122 (obovata).

R. Rueda 414 (lanuginosa); 525 (obovata).

R. Vásquez 9172 (insculpta); 10977 (insculpta); 5769 (obovata); 8604 (obovata); 23907 (obovata); 36497 (obovata).

R. Zúñiga 327 (obovata).

R.A.X. Borges 825 (insignis).

R.C. Monteiro da Costa 255 (obovata).

R.C. Quevedo S. 51 (obovata).

R.J. Burnham 1493 (obovata).

R.J. Evans 2591 (obovata).

R.L. Fróes 27592 (obovata); 27594 (obovata); 30203 (obovata); 31360 (obovata); 32723 (obovata); 27592a (obovata).

R.L. Liesner 26180 (obovata).

R.S. Rodr. s.n. (obovata).

S. McDaniel 23739 (obovata).

S. Ruysschaert SRU 728 (obovata).

S. Sakagawa 435 (obovata).

S.A. Mori 17998 (obovata).

S.R. King 492 (insculpta).

S.R.M. Silva 62 (obovata).

Schwacke 3610 (insculpta).

St.G. Beck 5372 (obovata).

T. Guedes 14 (obovata).

T. van Andel 680 (obovata).

T. Wendt 3865 (obovata).

T.B. Croat 88873 (obovata).

U.N. Maciel 1899 (obovata).

V. Demuner 4481 (insignis).

W. Palacios 272 (insculpta); 9153 (obovata).

W. Rodrigues 2203 (insculpta); 1956 (obovata); 2059 (obovata); 3846 (obovata); 4635 (obovata); 1956A (obovata); 4444 (tomentosa).

W. Thomas 4974 (insculpta).

W.A. Haber 2204 (obovata); W.A. Haber 2211 (obovata).

W.A. Rodrigues 9707 (insculpta).

W.H. Lewis 9975 (insculpta); 14026 (insculpta); 14389 (insculpta).

W.S. Alverson 266 (insculpta).
